# Impact of trifluoromethyl and sulfonyl groups on the biological activity of novel aryl-urea derivatives: synthesis, in-vitro, in-silico and SAR studies

**DOI:** 10.1038/s41598-023-44753-9

**Published:** 2023-10-16

**Authors:** Farid M. Sroor, Karima F. Mahrous, Heba A. M. Abd El-Kader, Abdelmageed M. Othman, Nada S. Ibrahim

**Affiliations:** 1 Organometallic and Organometalloid Chemistry Department, National Research Centre, Cairo, 12622 Egypt; 2 Cell Biology Department, National Research Centre, Dokki, 12622 Egypt; 3 Microbial Chemistry Department, Biotechnology Research Institute, National Research Centre, Dokki, 12622 Egypt; 4 Department of Chemistry (Biochemistry Branch), Faculty of Science, Cairo University, Giza, Egypt

**Keywords:** Biochemistry, Cancer, Drug discovery, Chemistry

## Abstract

We designed and prepared a novel series of urea derivatives with/without sulfonyl group in their structures to investigate the impact of the sulfonyl group on the biological activity of the evaluated compounds. Antibacterial investigations indicated that derivatives **7**, **8**, **9**, and **11** had the most antibacterial property of all the compounds examined, their minimum inhibitory concentrations (MICs) determined against *B. mycoides*, *E. coli*, and *C. albicans*, with compound **8** being the most active at a MIC value of 4.88 µg/mL. Anti-cancer activity has been tested against eight human cancer cell lines; A549, HCT116, PC3, A431, HePG2, HOS, PACA2 and BJ1. Compounds **7**, **8** and **9** emerged IC_50_ values better than Doxorubicin as a reference drug. Compounds **7** and **8** showed IC_50_ = 44.4 and 22.4 μM respectively against PACA2 compared to Doxorubicin (IC_50_ = 52.1 μM). Compound **9** showed IC_50_ = 17.8, 12.4, and 17.6 μM against HCT116, HePG2, and HOS, respectively. qRT-PCR revealed the down-regulation of *PALB2* in compounds **7** and **15** treated PACA2 cells. Also, the down-regulation of *BRCA1* and *BRCA2* was shown in compound **7** treated PC3 cells. As regard A549 cells, compound **8** decreased the expression level of *EGFR* and *KRAS* genes. While compounds **7** and **9** down-regulated *TP53* and *FASN* in HCT116 cells. Molecular docking was done against *Escherichia coli* enoyl reductase and human Son of sevenless homolog 1 (SOS1) and the results showed the promising inhibition of the studied proteins.

## Introduction

**Figure 1:**
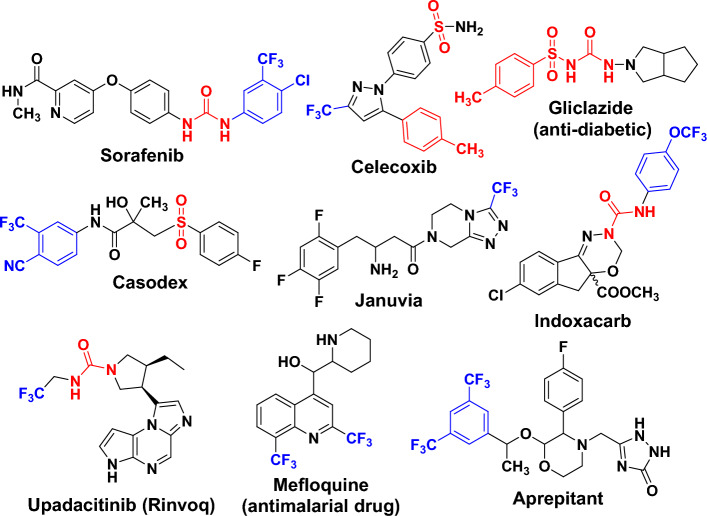
Marketed drugs containing trifluoromethyl group and/or aryl-urea derivatives.

The trifluoromethyl group (abbreviated as CF_3_), which is more bulky than the methyl group, is one of the most common lipophilic functional groups^1–3^. Due to the impact of the trifluoromethyl substituents on the electronic characteristics of the aromatic rings, the best-reported drugs containing aromatic trifluoromethyl substitution in their chemical structure. For example (as shown in Fig. 1), Prozac (anti-depressant fluoxetine), Emend (or Aprepitant as antiemetic drug), Celecoxib (arthritis medication and COX-2 inhibitor celecoxib), Casodex (or Bicalutamide as anticancer drug) and Januvia (used in the treatment of diabetes symptoms) having aromatic trifluoromethyl substitution^4–8^. Surprisingly, the presence of the trifluoromethyl substituent is common in increasing the potency by the formation of multipolar bindings with the carbonyl groups in the targeted protein^7^. On the other hand, the aryl-urea or sulfonyl-urea derivatives are of great interest in various areas of organic chemistry, coordination chemistry, and medicinal chemistry (Fig. 1) ^9–16^. It was reported the anticancer activity of several urea derivatives including *N*-nitroso-urea which is an alkylating agent drug^17,18^. Also, glibenclamide which is sulfonylurea proved to be a tumor growth inhibitor by inducing reactive oxygen species (ROS) followed by apoptosis of cancer cells^19^. Indeed, the combination of aromatic trifluoromethyl substitutions with urea moiety in one compound will increase the biological activity of the final product.

Antibiotic resistance (which refers to microorganisms' resistance to antimicrobial medicines) has made treating life-threatening bacterial infections much more difficult^20,21^. If the current rate of antimicrobial resistance continues, it is postulated that if no steps were taken, drug-resistant infections will be responsible for 10 million deaths worldwide per year by 2050, exceeding cancer deaths^22^. Antibiotic use is a major contributor to antibiotic resistance. In communities, primary care facilities, nursing homes, and particular hospitals, as well as across nations, the link between antibiotic usage and resistance has been widely documented^23,24^. There are various difficulties in quantifying the illness burden associated with infections caused by antibiotic-resistant bacteria. For example, sampling and microbiological protocols for testing isolates, data-gathering processes, and surveillance system architecture may differ between and within nations. Furthermore, understanding of the clinical and public health repercussions of antibiotic-resistant bacterial infections in people is relatively limited. In particular, the scientific debate is raging over the best epidemiological research design and statistical inference methodologies for making realistic estimates of adverse clinical outcomes caused by antibiotic-resistant bacterial infections^21,25,26^. National and international agencies have devised and are implementing action plans to slow the spread of antimicrobial resistance. Research and innovation, infection control practices, stewardship, and surveillance are the four basic principles of these action plans^22,23,27^.

Antibiotic resistance frequently develops as a result of extended usage, and it typically promotes the growth of resistant bacterial isolates while inhibiting the growth of susceptible germs. Most of the time, continual antibiotic pressure leads to resistance not only to the antibiotic in question but also to other antibiotics in the same class^20,28^. Bacterial antibiotic resistance mechanisms are mainly classified into three basic pathways: inhibition of antibiotic binding to the target site through exclusion techniques, modification or destruction of the antibiotic molecule, and mutation of the antibiotic target site. Furthermore, bacteria typically acquire resistance genes by transformation through getting resistant genes from their surroundings, transduction through transferring resistant genes from bacteriophages, and bacterial conjugation via resistant gene transfer between resistant bacterial strains^29,30^. As antibiotic-resistant bacteria become more common, replacements for antibiotics should indeed be recognized. Some of the proposed alternatives include antibiotic framework alteration, combinational administration of drugs, antibiotic-adjuvant hybrids, as well as the use of biopharmaceuticals^20^.

Consequently and in continuation of our efforts to design and develop biologically active organic compounds^31–36^, in the current study we decided to design and synthesize a new series of aryl-urea derivatives containing trifluoromethyl substitutions under mild and metal-free conditions to increase the biological profile of the final products as antimicrobial and anti-cancer agents.

## Materials and methods

### Chemistry

All reactions were carried out in aerobic conditions at room temperature. Acetonitrile was distilled and kept under an inert atmosphere. All glassware was oven-dried at 120 °C for at least 24 h before use. The starting materials of the primary amines **1**, **2**, **3**, **4** and **5** and the isocyanate derivatives, 4-tolyl sulfonyl isocyanate (**6**) and 4-tolyl isocyanate (**12**) were purchased from Aldrich and used as received. All melting points are uncorrected and measured using Electro‐Thermal IA 9100 apparatus (Shimadzu, Japan). The Infrared spectra were recorded as potassium bromide pellets on a JASCO spectrophotometer between 4000 cm^−1^ and 400 cm^−1^. ^1^H NMR and ^13^C NMR spectra were recorded in deuterated dimethyl sulfoxide (DMSO-d_6_) on a Brucker spectrometer (400 MHz) at 25 °C. The chemical shifts were expressed as part per million (*δ* values, ppm) against TMS as an internal reference. Microanalyses were operated using Mario Elmentar apparatus, Organic Microanalysis Unit, National Research Centre (NRC), Cairo, Egypt.

### General procedure for the synthesis of urea derivatives (7–11 and 13–17)

4-Tolyl sulfonyl isocyanate (**6**) (0.01 mol) or 4-tolyl isocyanate (**12**) (0.01 mol) was added to a solution of the primary amines (**1**, **2**, **3**, **4**, or **5**) (0.01 mol) in acetonitrile while stirring at room temperature. The reaction mixture was stirred to the desired time. The completion of reactions was monitored by TLC on silica gel-coated aluminum sheets. The obtained precipitate was filtered off, washed with cold acetonitrile and dried well, then recrystallized from ethyl acetate/acetonitrile (3:1) to give:

#### 4-Methyl-*N*-[(4-{trifluoromethyl}phenyl)carbamoyl]benzenesulfonamide (7)

Compound **7** precipitated after 15 min as a white solid; m.p. 194–195 °C. IR (KBr): ν (cm^-1^) 3318, 3286 (NH), 1682 (C=O), 1341, 1167 (SO_2_). ^1^H NMR (DMSO-d_6_): *δ* (ppm) 2.35 (s, 3H, CH_3_), 7.26–7.85 (m, 9H, Ar + NH), 9.23 (br, 1H, NH). ^13^C NMR (DMSO-d_6_): *δ* (ppm) 21.4, 113.5, 119.3, 126.2, 126.6, 126.7, 128.1, 129.8, 137.4, 142.0, 142.4, 144.5, 150.0, 152.7. Anal. Calcd. for C_15_H_13_F_3_N_2_O_3_S (358.34): C, 50.28; H, 3.66; N, 7.82. Found: C, 50.36; H, 3.58; N, 7.77.

#### 4-Methyl-*N*-[(4-{trifluoromethoxy}phenyl)carbamoyl]benzenesulfonamide (8)

Compound **8** precipitated after 7 min as a white solid; m.p. 183–184 °C. IR (KBr): ν (cm^−1^) 3322, 3284 (NH), 1681 (C=O), 1343, 1165 (SO_2_). ^1^H NMR (DMSO-d_6_): *δ* (ppm) 2.33 (s, 3H, CH_3_), 7.20–7.86 (m, 9H, Ar + NH), 9.03 (br, 1H, NH). ^13^C NMR (DMSO-d_6_): *δ* (ppm) 21.5, 115.0, 121.0, 122.0, 122.4, 126.2, 127.2, 129.4, 130.0, 137.8, 144.1, 144.4, 148.2, 150.0. Anal. Calcd. for C_15_H_13_F_3_N_2_O_4_S (374.33): C, 48.13; H, 3.50; N, 7.48. Found: C, 48.26; H, 3.59; N, 7.54.

#### *N*-[(3,5-bis{Trifluoromethyl}phenyl)carbamoyl]-4-methylbenzenesulfonamide (9)

Compound **9** precipitated after 20 min as a white solid; m.p. 172–174 °C. IR (KBr): ν (cm^−1^) 3323, 3281 (NH), 1685 (C=O), 1344, 1166 (SO_2_). ^1^H NMR (DMSO-d_6_): *δ* (ppm) 2.34 (s, 3H, CH_3_), 7.01–8.0 (m, 8H, Ar + NH), 9.50 (br, 1H, NH). ^13^C NMR (DMSO-d_6_): *δ* (ppm) 21.4, 100.0, 107.6, 113.4, 122.3, 125.6, 128.0, 129.5, 130.1, 131.6, 136.4, 140.9, 142.4, 144.5, 150.4, 151.0. Anal. Calcd. for C_16_H_12_F_6_N_2_O_3_S (426.33): C, 45.08; H, 2.84; N, 6.57. Found: C, 45.16; H, 2.99; N, 6.74.

#### *N*-[(3,5-Dimethoxyphenyl)carbamoyl]-4-methylbenzenesulfonamide (10)

Compound **10** precipitated after 35 min as a white solid; m.p. 160–161 °C. IR (KBr): ν (cm^−1^) 3328, 3285 (NH), 1688 (C=O), 1349, 1168 (SO_2_). ^1^H NMR (DMSO-d_6_): *δ* (ppm) 2.36 (s, 3H, CH_3_), 3.48 (s, 6H, OCH_3_), 6.75–6.79 (m, 2H, Ar), 6.98 (s, 1H, Ar), 7.38–7.40 (m, 2H, Ar), 7.78–7.80 (m, 2H, Ar), 8.60 (br, 1H, NH), 10.51 (br, 1H, NH). ^13^C NMR (DMSO-d_6_): *δ* (ppm) 21.6, 56.0, 105.0, 111.5, 112.7, 126.1, 128.2, 130.0, 131.9, 137.8, 141.0, 142.1, 144.3, 145.5, 149.0, 149.8. Anal. Calcd. for C_16_H_18_N_2_O_5_S (350.39): C, 54.85; H, 5.18; N, 8.00. Found: C, 54.92; H, 5.79; N, 8.14.

#### 4-Methyl-*N*-[(2-{trifluoromethyl}phenyl)carbamoyl]benzenesulfonamide (11)

Compound **11** precipitated after 20 min as a white solid; m.p. 140–141 °C. IR (KBr): ν (cm^−1^) 3326, 3281 (NH), 1683 (C=O), 1340, 1164 (SO_2_). ^1^H NMR (DMSO-d_6_): *δ* (ppm) 2.33 (s, 3H, CH_3_), 7.24–7.82 (m, 8H, Ar), 8.23 (br, 1H, NH), 11.31 (br, 1H, NH). ^13^C NMR (DMSO-d_6_): *δ* (ppm) 21.3, 115.5, 117.3, 125.8, 126.2, 126.4, 127.9, 129.8, 130.1, 133.5, 137.3, 142.4, 144.6, 146.7, 150.2. Anal. Calcd. for C_15_H_13_F_3_N_2_O_3_S (358.34): C, 50.28; H, 3.66; N, 7.82. Found: C, 50.42; H, 3.74; N, 7.94.

#### 1-(p-Tolyl)-3-[4-(trifluoromethyl)phenyl]urea (13)

Compound **13** precipitated after 13 min as a white solid; m.p. 263–265 °C. IR (KBr): ν (cm^−1^) 3320, 3279 (NH), 1681 (C=O). ^1^H NMR (DMSO-d_6_): *δ* (ppm) 2.21 (s, 3H, CH_3_), 7.06 (d, 2H, *J* = 8 Hz, Ar), 7.31 (d, 2H, *J* = 8 Hz, Ar), 7.60–7.63 (m, 4H, Ar), 8.65 (br, 1H, NH), 9.01 (br, 1H, NH). ^13^C NMR (DMSO-d_6_): *δ* (ppm) 20.8, 118.1, 118.3, 119.2, 125.0, 123.4, 126.6, 129.7, 129.6, 131.7, 137.2, 138.1, 144.1, 152.8. Anal. Calcd. for C_15_H_13_F_3_N_2_O (294.28): C, 61.22; H, 4.45; N, 9.52. Found: C, 61.32; H, 4.51; N, 9.64.

#### 1-(p-Tolyl)-3-[4-(trifluoromethoxy)phenyl]urea (14)

Compound **14** precipitated after 5 min as a white solid; m.p. 240–241 °C. IR (KBr): ν (cm^−1^) 3322, 3282 (NH), 1686 (C=O). ^1^H NMR (DMSO-d_6_): *δ* (ppm) 2.20 (s, 3H, CH_3_), 7.06 (d, 2H, *J* = 8 Hz, Ar), 7.25–7.30 (m, 4H, Ar), 7.52 (d, 2H, *J* = 8 Hz, Ar), 8.57 (br, 1H, NH), 8.80 (br, 1H, NH). ^13^C NMR (DMSO-d_6_): *δ* (ppm) 20.8, 109.1, 118.9, 119.0, 120.1, 121.5, 122.2, 125.1, 129.7, 131.4, 137.4, 140.2, 143.0, 153.0. Anal. Calcd. for C_15_H_13_F_3_N_2_O_2_ (310.28): C, 58.07; H, 4.22; N, 9.03. Found: C, 58.12; H, 4.35; N, 9.14.

#### 1-[3,5-Bis(trifluoromethyl)phenyl]-3-(p-tolyl)urea (15)

Compound **15** precipitated after 3 h as a white solid; m.p. 204–205 °C. IR (KBr): ν (cm^−1^) 3328, 3291 (NH), 1685 (C=O). ^1^H NMR (DMSO-d_6_): *δ* (ppm) 2.20 (s, 3H, CH_3_), 7.03–7.05 (m, 2H, Ar), 7.29–7.31 (m, 3H, Ar), 8.09 (s, 1H, Ar), 8.45 (s, 1H, Ar), 8.83 (br, 1H, NH), 9.30 (br, 1H, NH). ^13^C NMR (DMSO-d_6_): *δ* (ppm) 20.8, 115.1, 118.4, 118.8, 119.6, 122.3, 125.3, 129.6, 129.7, 131.0, 132.0, 136.9, 137.7, 142.5, 153.1, 153.2. Anal. Calcd. for C_16_H_12_F_6_N_2_O (362.28): C, 53.05; H, 3.34; N, 7.73. Found: C, 53.06; H, 3.55; N, 7.84.

#### 1-[3,5-Dimethoxyphenyl]-3-(p-tolyl)urea (16)

Compound **16** precipitated after 30 s as a white solid; m.p. 188–190 °C. IR (KBr): ν (cm^−1^) 3325, 3280 (NH), 1687 (C = O). ^1^H NMR (DMSO-d_6_): *δ* (ppm) 2.20 (s, 3H, CH_3_), 3.67–3.72 (m, 6H, OCH_3_), 6.82 (s, 2H, Ar), 7.06 (d, 2H, *J* = 8 Hz, Ar), 7.16 (s, 1H, NH), 7.30 (d, 2H, *J* = 8 Hz, Ar), 8.41–8.43 (m, 2H, Ar + NH). ^13^C NMR (DMSO-d_6_): *δ* (ppm) 20.8, 52.5, 55.3, 55.9, 104.4, 110.6, 113.0, 118.8, 129.7, 131.0, 134.0, 137.8, 144.5, 149.3, 153.3. Anal. Calcd. for C_16_H_18_N_2_O_3_ (286.33): C, 67.12; H, 6.34; N, 9.78. Found: C, 67.19; H, 6.39; N, 9.84.

#### 1-(p-Tolyl)-3-[2-(trifluoromethyl)phenyl]urea (17)

Compound **17** precipitated after 3 h as a white solid; m.p. 262–263 °C. IR (KBr): ν (cm^−1^) 3325, 3287 (NH), 1684 (C=O). ^1^H NMR (DMSO-d_6_): *δ* (ppm) 2.20 (s, 3H, CH_3_), 7.04–7.07 (m, 3H, Ar), 7.29–7.32 (m, 3H, Ar), 7.60–7.63 (m, 1H, Ar), 7.98–8.00 (m, 1H, Ar), 8.46 (s, 1H, NH), 9.25 (br, 1H, NH). ^13^C NMR (DMSO-d_6_): *δ* (ppm) 20.8, 118.7, 118.8, 124.5, 126.0, 126.1, 129.0, 129.5, 129.7, 131.5, 133.2, 137.4, 137.7, 153.0, 153.2. Anal. Calcd. for C_15_H_13_F_3_N_2_O (294.28): C, 61.22; H, 4.45; N, 9.52. Found: C, 61.34; H, 4.56; N, 9.61.

### Antimicrobial activity evaluation

To test the antimicrobial property of prepared substances, *Bacillus mycoides* (Gram-positive) bacterium, *Escherichia coli* (Gram-negative), and *Candida albicans* (non-filamentous fungus) have been used as model microbes. Microorganisms were cultivated and kept at pH 7.0 in a nutritional agar medium (70148 Nutrient agar, Fluka, Spain). The antimicrobial activity of the produced substances was evaluated by using the agar well diffusion procedure as regards: Every hardened nutrient agar plate received 100 µL (4 × 10^7^ CFU) from every 24 h re-activated culture. The infected hardened plates were punctured with 15 mm holes to accommodate a quantity of 200 µL per each dissolved component (10 mg/mL) in DMSO. The seeded cultivation dishes with test solutions were incubated for 24 h at 37 °C, and the resulting clear zones have been reported. The minimum inhibitory concentration (MIC) of constituents **7**, **8**, **9**, and **11** was distinguished by a serial dilution method employing DMSO as a solvent. A range of doses from 10 to 0.00488 mg/mL were used. The MIC of every component along each microbe was specified as the lowest concentration of that component capable of inhibiting the development of the given microbe^14,37^.

### Evaluation of cell proliferation by MTT assay

The human lung carcinoma (A549), colon cancer (HCT116), prostate cancer (PC3), skin cancer (A431), hepatocellular carcinoma cell line (HePG2), osteosarcoma cell line (HOS), pancreatic cancer cell line (PACA2) and normal skin fibroblast cell line (BJ1) were purchased from American Tissue Culture Collection (Rockville, MD, USA). HCT116, A549, HePG2, A431, PACA2 and MCF7 were maintained in DMEM media, while PC3 was in RPMI-1640 media (Lonza, Biowahittkar, Belgium). The human normal BJ1 was maintained in DMEM-F12 media. The used media were supplemented with a 1% antibiotic–antimycotic mixture (10,000 µg/mL streptomycin sulfate, 10,000 U/mL potassium penicillin, 1% l-glutamine, 25 µg/mL amphotericin B and 10% fetal bovine serum (FBS) (Biowest, USA). The percentage of viable cells was determined using MTT [3-(4,5-dimethylthiazol-2-yl)-2,5-diphenyltetrazolium bromide] (Bio Basic Canada Inc., Canada). In brief, about 10^4^ cells/ well were seeded into a 96-well plate and incubated at 37 °C for 24 h to be allowed to adhere. Then, the prepared compounds were added at different concentrations of 100, 50, 25 and 12.5 µg/mL to the cell monolayer in triplicate and incubated at 37 °C for 48 h. After that, the medium was replaced with another fresh medium, and 40 µL MTT (2.5 µg/mL) was added to each well and kept for a further 4 h. At last, 200 µL of 10% sodium dodecyl sulfate (SDS) was added to each well and incubated at 37 °C overnight to allow the reaction to be stopped and break up the formed formazan crystals. Subsequently, the quantity of formazan product was detected at a wavelength of 595 nm with a reference wavelength of 620 nm using a microplate reader (Bio-Rad Laboratories, model 3360, USA). Doxorubicin (Adriamycin) was used as a positive control. The vehicle was dimethyl sulfoxide (DMSO), which is used for dissolving the prepared compounds and the final concentration of it was less than 0.2%. IC_50_ was calculated using the Prism software program (Graph Pad software incorporated, version 3).

### Gene expression analysis

#### Quantitative real-time PCR method

RNA was extracted from pancreas, prostate, lung and liver cell lines using a total RNA purification kit (Qiagen, Hilden, Germany) according to the manufacturer's instructions. An aliquot of RNA was diluted in RNase-free water to estimate RNA quantity. Aliquots were used immediately for reverse transcription (RT). cDNA synthesis was performed on extracted RNA, which was treated with DNase (Invitrogen, Germany) to remove any possible DNA contamination. The DNase-treated RNA was reverse transcribed into first-strand cDNA using the RevertAid First Strand cDNA Synthesis kit (Fermantas) according to the manufacturer's instructions. Determination of the pancreas, prostate, lung and liver cell line cDNA copy number was carried out using StepOne™ Real-Time PCR System from Applied Biosystems (Thermo Fisher Scientific, Waltham, MA USA). Gene expressions were detected by real-time PCR, which was performed using the Rotor-Gene Q system (Qiagen Company). A 25 µL reaction mixture consisted of 12.5 µL SYBR® Premix Ex TaqTM (TaKaRa, Biotech. Co. Ltd.), 0.5 µL of each primer (10 PMole) (Table 1), 1 µL cDNA (50 ng) and 10.5 µL RNase free water. The optimum amplification conditions were chosen empirically according to each tested gene. Generally, the amplification conditions include initial incubation (95.0 °C for 3 min), then 40 cycles of amplification with denaturation (95.0 °C for 15 s), annealing (55.0 °C for 30 s) and extension (72.0 °C for 30 s) steps then 71 cycles which started at 60.0 °C and then increased about 0.5 °C every 10 s up to 95.0 °C. Mean cycle threshold (Ct) values of triplicate samples are used for analysis. The Ct value indicates the fractional cycle number at which the amount of amplified target reaches a fixed threshold. Data from real-time PCR were analyzed using 2^−∆∆Ct^ method^38–40^. Data were represented as the fold change in target gene expression normalized to a House-Keeping gene (HKG) and relative to the control. *β-actin* was used as a HKG to normalize input RNA amount, RNA quality and reverse transcription efficiency.

**Table 1:** Primers sequence used for *qRT-PCR*.

Gene	Primer sequence	GenBank (accession no)
*PALB2*	F: TGGGTGTGATGCTGTACTGT R: CCAGCCAGCAAATGAGAGTC	EU831697.1
*CDKN2A*	F: GGGTCCCAGTCTGCAGTTAA R: TGAACCACGAAAACCCTCAC	U38945.1
*BRCA1*	F: TGAAGAAAGAGGAACGGGCT R: TGGCTCCCATGCTGTTCTAA	KJ901305.1
*BRCA2*	F: GGGATGACACAGCTGCAAAA R: TGGGCCTTAACAGCATACCA	GU014835.1
*EGFR*	F: AGGTGAAAACAGCTGCAAGG R: AGGTGATGTTCATGGCCTGA	KJ904454.1
*KRAS*	F: AGTGCCTTGACGATACAGCT R: CCTCCCCAGTCCTCATGTAC	AF493917.1
*TP53*	F: TGGCCATCTACAAGCAGTCA R: GGTACAGTCAGAGCCAACCT	KJ897694.1
*FASN*	F: GCCTTTGAAATGTGCTCCCA R: GTGAACTGCTGCACGAAGAA	BC014634.2
*β-actin*	F: CATGGAATCCTGTGGCATCC R: CACACAGAGTACTTGCGCTC	HQ154074.1

#### DNA damage using the comet assay

According to the method of Olive et al.^41^ after the trypsin treatment of pancreas, prostate, lung and liver cancer cell lines to produce a single cell suspension. This suspension was stirred for 5 min and filtered. 100 μL of cell suspension was mixed with 600 μL of low-melting agarose (0.8% in PBS). 100 μL of this mixture was spread on pre-coated slides which immersed in lysis buffer (0.045 M TBE, pH 8.4, containing 2.5% SDS) for 15 min. The slides were placed in an electrophoresis chamber containing the same TBE buffer, but devoid of SDS. The electrophoresis conditions were 2 V/cm for 2 min and 100 mA. Staining with ethidium bromide 20 μg/mL. at 4 °C.

#### Comet capture and analysis

A total of 100 randomly captured comets from each slide were examined at 400 × magnification using a fluorescence microscope connected to a CCD camera to an image analysis system [comet 5 image analysis software developed by Kinetic Imaging, Ltd. (Liverpool, UK)]. A computerized image analysis system acquires images, computes the integrated intensity profiles for each cell, estimates the comet cell components and then evaluates the range of derived parameters. To quantify the DNA damage tail length (TL), the percentage of migrated DNA (Tail DNA%) and tail moment (TM) were evaluated. The non-overlapping cells were randomly selected and were visually assigned a score on an arbitrary scale of 0–3 (i.e., class 0 = no detectable DNA damage and no tail; class 1 = tail with a length less than the diameter of the nucleus; class 2 = tail with length between 1 × and 2 × the nuclear diameter; and class 3 = tail longer than 2 × the diameter of the nucleus) based on perceived comet tail length migration and relative proportion of DNA in the nucleus^42^.

#### DNA fragmentation assay

DNA fragmentation in the pancreas, prostate, lung and liver cancer cell lines was carried out according to Yawata^43^ with some modifications. Briefly, after 24 h of exposure of pancreas, prostate, lung and liver cancer cell lines to the evaluated compounds in different Petri dishes (60 × 15 mm, Greiner), the cells were trypsinized, suspended and homogenized in 1 ml of medium. Then, the cells were centrifuged for 10 min at 800 rpm. After the centrifugation step, the low molecular weight genomic DNA was extracted as described in Yawata^43^. Approximately, 1 × 10^6^ cells were seeded and treated with the IC_50_ concentration of evaluated compounds. All the cells (including floating cells) were collected by the treatment with trypsin and washed with Dulbecco`s Phosphate Buffered Saline (PBS). On ice, the cells were lysed with the lysis buffer containing 150 mM NaCl, 10 mM Tris (pH 7.4), 5 mM ethylenediaminetetraacetic acid (EDTA), and 0.5% Triton X-100 for 30 min. The lysates were mixed using a vortex and then centrifuged for 20 min at 10,000×*g*. The damaged DNA in the supernatant was extracted with an equal volume of neutral phenol: chloroform: isoamyl alcohol mixture (25:24:1). The percentage of fragmented DNA was then analyzed using gel electrophoresis on 2% agarose gel including 0.1 µg/mL ethidium bromide.

#### Molecular docking study

Molecular operating environment (MOE 2009.10) and BIOVIA Discovery Studio programs were used to perform the molecular docking study^44^. The mode of interaction between the evaluated compounds **8** and **9** and the active site of the studied proteins (*Escherichia coli* enoyl reductase and human SOS1) was visualized in 2-dimensional and 3-dimensional states using the BIOVIA Discovery Studio program. The studied proteins were downloaded from the protein data bank (www.pdb.org), where the protein codes were (1C14 and 6SCM) respectively. The co-crystalized standard ligand complexed with the studied proteins were (Triclosan and BI-3406) respectively. The target compounds **8** and **9** were drawn using the MOE builder interface and then subjected to local and global energy minimization using the included MOPAC. The energy of the target compounds was calculated by performing the systematic conformational search where RMS gradient and RMS distance were set as default at 0.01 kcal/mole and of 0.1A^o^ respectively. The lowest value of energy for the target compounds was selected to be used in the next docking step. Several modifications were done on the selected proteins for the subsequent docking studies which included the following steps: (a) The hydrogen atoms were added to the selected target proteins; (b) The co-crystalized standard ligand molecule was removed from the protein active site; (c) The active site was selected using MOE alpha site finder and dummy atoms were prepared from the obtained alpha spheres; (d) The prepared model was then subjected to the docking step to predict the ligand–protein binding interactions at the active site.

## Results and discussion

### Chemistry

Under simple and free-metal condition reaction, the treatment of 4-tolyl sulfonyl isocyanate (**6**) with primary amines of 4-(trifluoromethyl)aniline (**1**), 4-(trifluoromethoxy)aniline (**2**), 3,5-bis(trifluoromethyl)aniline (**3**), 3,5-dimethoxy aniline (**4**), and 2-(trifluoromethyl)aniline (**5**) in acetonitrile at room temperature afforded the corresponding 4-tolyl sulfonylurea derivatives **7**–**11**, respectively. Likewise, the reaction of 4-tolylisocyanate (**12**) with the same previous amines afforded 4-tolylurea derivatives** 13**–**17**, respectively.

**Figure 2:**
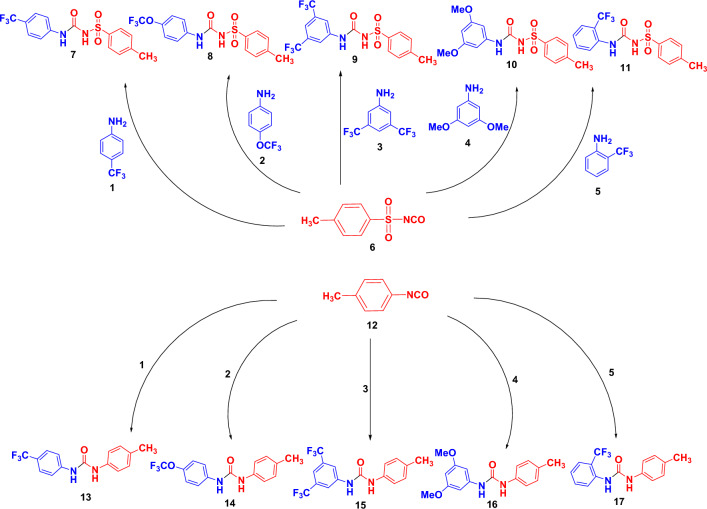
Synthesis of 4-tolyl sulfonylurea (**7**–**11**) and 4-tolylurea derivatives (**13**–**17**). All the reactions were carried out in acetonitrile at room temperature.

All compounds **7**–**11** and **13**–**17** were prepared in good to excellent yield. The chemical structures of the newly synthesized compounds **7**–**11** and **13**–**17** were approved by IR, ^1^H-NMR, ^13^C-NMR, and elemental analysis. Compound **9** (Fig. 2) will be discussed in detail as a represented example of sulfonyl-urea derivatives **7**–**11**. In the ^1^H-NMR spectrum (400 MHz, DMSO-*d*_*6*_) the protons of the aromatic rings and one NH group were assigned at range *δ* 7.01–8.0 ppm as multiplet signals, the other NH group which attached to the sulfonyl group was assigned at *δ* 9.50 ppm as a singlet signal. The protons of the methyl group were assigned at *δ* 2.34 ppm as a singlet signal. Likewise, all the expected number of carbon signals was observed in the ^13^C-NMR spectrum of **9**. In the IR, the NH group was assigned at ν 3323 and 3281 cm^−1^, while the C=O was attributed at ν 1685 cm^−1^ and the sulfonyl group (SO_2_) was assigned at ν1344 and 1166 cm^−1^. On the other hand, compound** 13** (Fig. 2) will be explained as a represented example of aryl-urea derivatives **13**–**17**. The ^1^H-NMR spectrum of **13** showed the NH groups as singlet in the downfield zone at *δ* 8.65 and 9.01 ppm, while the methyl group was assigned as expected at *δ* 2.21 ppm. The aromatic protons of 4-tolyl ring were assigned at *δ* 7.06 and 7.31 ppm as a doublet with coupling constant (*J* = 8 Hz), while the aromatic protons of the 4-trifluoromethyl phenyl ring were observed at *δ* 7.61 ppm as a multiplet signal. In the ^13^C-NMR spectrum, all the carbons were observed at their expected chemical shifts. The IR spectrum of 13 visualized the NH group at ν 3320 and 3279 cm^−1^ and the carbonyl group at ν 1681 cm^−1^.

### Antimicrobial activity

#### Antimicrobial activity testing utilizing the agar diffusion method

To assess the efficacy of the prepared formulations as antimicrobial agents, different microbial populations, *B. mycoides*, *E. coli*, and *C. albicans*, have been chosen as examples for Gram-positive, Gram-negative bacteria, and non-filamentous fungi, respectively. To evaluate the antimicrobial properties of the produced substances against the preceding indicated microbes, the agar well diffusion technique was used. The specific antimicrobial agent's activity, and therefore its efficacy as an antiseptic agent, is dependent mostly on the cell structure of microbial species, along with the main ingredients of the evaluated chemical^14,45,46^.

The antimicrobial efficacy of the generated components (compounds **7**–**17**) was displayed in Table 2. The acquired findings demonstrated the disparity in action and responsiveness between each of the various evaluated chemicals and microbes (Table 2). Compound **9** had the maximum activity versus *B. mycoides* and *C. albicans* (41 mm), trailed by compound **8**, which exhibited comparable results against *B. mycoides*, and *C. albicans* (37 and 38 mm, respectively). Compounds **7** and **11** also showed significant antimicrobial properties against investigated microbial species, with greater values in opposition to *C. albicans*. Other substances, on the other hand, had reduced antimicrobial properties against all evaluated microbes, with compounds **13**, **14**, **16**, and **17** exhibiting the worst activity. As a side note, *E. coli*, as a typical Gram-negative bacterium, demonstrated the highest resilience amongst studied microorganisms, which might be attributed to the composition of its cell wall (Table 1). Because of their significant antibacterial properties, compounds **7**, **8**, **9**, and **11** have been selected to determine their MIC values to stop the proliferation of tested microorganisms.

**Table 2:** Antimicrobial activity assessment of compounds **7**–**17** using agar diffusion technique.

Compound	Inhibition zones diameter (mm)		
	*B. mycoides*	*E. coli*	*C. albicans*
***7***	*36* ± *1.25*	*30* ± *0.74*	*37* ± *1.20*
***8***	*37* ± *0.23*	*29* ± *0.44*	*38* ± *0.67*
***9***	*41* ± *0.0*	*37* ± *0.74*	*41* ± *0.21*
**10**	24 ± 0.87	22 ± 0.39	21 ± 0.32
***11***	*31* ± *0.52*	*27* ± *0.58*	*32* ± *0.17*
**13**	16 ± 0.41	18 ± 1.84	18 ± 0.66
**14**	15 ± 0.25	17 ± 0.81	17 ± 0.27
**15**	23 ± 0.35	24 ± 0.68	24 ± 1.22
**16**	16 ± 0.97	16 ± 0.35	16 ± 0.95
**17**	18 ± 0.0	16 ± 0.54	15 ± 0.42
DMSO (control)	0	0	0
Colstin 10 mcg	0	0	0
Tobramycin 10 mcg	10 ± 0.31	13 ± 0.22	12 ± 0.61
Gentamicin 10 mcg	11 ± 0.43	12 ± 0.18	12 ± 0.89
Ampicillin 10 mcg	12 ± 0.93	20 ± 1.05	14 ± 0.25
Erythromycin 15 mcg	17 ± 0.15	29 ± 2.13	25 ± 0.00

Significant values are in italics.

200 μL of dissolved compounds (10 mg/mL) in DMSO were applied to 15 mm holes prepared in the inoculated agar plates. Culture plates were incubated overnight at 37 °C.

**Figure 3:**
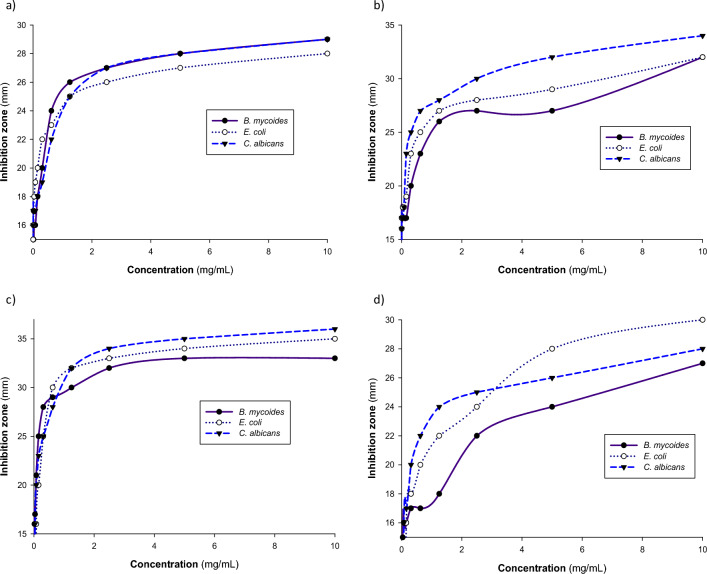
Effect of different concentrations of compounds (**a**) **7** (**b**) **8** (**c**) **9** and (**d**) **11** on their antimicrobial activity. Each sample (150 µL) at different concentrations was dissolved in DMSO and added to a 15 mm agar well. Inoculum (4 × 10^7^ CFU) was added to each 20 cm plate (150 mL of 70148 nutrient agar, Fluka), then the plates were incubated overnight at 37 °C.

The serial dilution procedure was applied to determine the MICs of the most powerful synthesized compounds. The results provided for determining the MIC values of compounds **7**, **8**, **9**, and **11** have been included in Table 3. The antimicrobial activity of evaluated compounds was shown to be proportionate to their dosages (Table 3 and Fig. 3). Furthermore, the MIC values of every compound against evaluated microorganisms are affected by both the cell structure of the microbes, as well as the overall constitution of the examined compound^45^. A compound with a lower MIC value has a greater potential to inhibit the development of the designated microbe^14^. Compounds **7** and **8** have a minimum MIC level (≥ 4.88 µg/mL) versus different classes of microorganisms, with a MIC value of 4.88 µg/mL in all cases except the case of compound **7** against *B. mycoides* (9.75 µg/mL). On the other hand, the highest MIC value was recorded with compound **11** against *E. coli* (Table 4).

**Table 3:** Effect of different concentrations of compounds **7**, **8**, **9** and **11** on their antimicrobial activity.

Concentration (mg/mL)	Inhibition zone (mm)											
	*B. mycoides*				*E. coli*				*C. albicans*			
	**7**	**8**	**9**	**11**	**7**	**8**	**9**	**11**	**7**	**8**	**9**	**11**
10.0	29	32	33	27	28	32	35	30	29	34	36	28
5.00	28	27	33	24	27	29	34	28	28	32	35	26
2.50	27	27	32	22	26	28	33	24	27	30	34	25
1.25	26	26	30	18	25	27	32	22	25	28	32	24
0.625	24	23	29	17	23	25	30	20	22	27	28	22
0.3125	20	20	28	17	22	23	25	18	19	25	25	20
0.1563	18	17	25	16	20	19	20	16	18	23	23	17
0.0781	16	17	21	16	19	18	16	0	17	18	20	16
0.0391	16	17	17	15	18	18	0	0	17	17	16	0
0.0195	15	17	16	0	17	17	0	0	17	17	0	0
0.00975	15	17	0	0	16	17	0	0	17	17	0	0
0.00488	0	17	0	0	15	16	0	0	16	16	0	0
0 (DMSO)	0	0	0	0	0	0	0	0	0	0	0	0

Each sample (150 µL) at different concentrations was dissolved in DMSO and added to a 15 mm agar well. Inoculum (4 × 10^7^ CFU) was added to each 20 cm plate (150 mL of 70,148 nutrient agar, Fluka), and then the plates were incubated overnight at 37 °C.

**Table 4:** Minimum inhibition concentration (MIC) of compounds **7**, **8**, **9** and **11** preparations against some microorganisms.

Compound	Minimum inhibition concentration (µg/mL)		
	*B. mycoides*	*E. coli*	*C. albicans*
**7**	9.75	4.88	4.88
**8**	4.88	4.88	4.88
**9**	19.5	78.1	39.1
**11**	39.1	156.3	78.1

### Anti-cancer activity

#### Primary screening

Compounds **7–11** and **13–17** were screened against seven human cancer cell lines, lung carcinoma (A549), colon cancer (HCT116), prostate cancer (PC3), human skin cancer (A431), hepatocellular carcinoma cell line (HePG2), osteosarcoma cell line (HOS), pancreatic cancer cell line (PACA2), at 100 μg/mL. Also, the compounds were evaluated against the normal skin fibroblast cell line (BJ1) for comparison purposes. As shown in Table 5, compound **7** exhibited 100% mortality against all the evaluated cancer cell lines except A431 which showed 55.4% mortality. Compound **8** exhibited 100% mortality against A549, HCT116, PC3, HePG2 and PACA2, and more than 50% mortality against HOS. Also, 100% mortality was exhibited for compound **9** against HCT116, A431, HePG2, and HOS (Table 5). Compound **11** exhibited more than 75% mortality against A549, HCT116, PC3, and PACA2, while compound **13** showed 100% mortality against only HePG2 and about 74.3% mortality against HOS. Regarding compound **14**, 100% mortality was exhibited against HOS and 65.3% mortality against HePG2. Compound **15** exhibited more than 75% mortality against PC3, HOS, and PACA2, and more than 50% mortality against A549. More than 50% mortality was exhibited for compounds **10**, **16**, and **17** against PACA2, (HOS and PACA2), (PC3, HOS and PACA2) respectively. So, the evaluated compounds against these selected cell lines were subjected to secondary screening to determine their IC_50_ and selectivity index values.

**Table 5:** Mortality (%) of cancer and normal cell lines at 100 μg/mL.

Comp	A549	HCT116	PC3	A431	HePG 2	HOS	PACA2	BJ1
7	*100*	*100*	*100*	55.4 ± 1.25	*100*	*100*	*100*	12.3 ± 1.12
8	*100*	*100*	*100*	27.5 ± 0.64	*100*	57.2 ± 0.29	*100*	6.2 ± 1.54
9	–	*100*	–	*100*	*100*	*100*	–	28 ± 0.61
10	34.2 ± 1.20	37.5 ± 1.12	35.2 ± 0.89	23.5 ± 0.97	–	12.3 ± 1.21	55.4 ± 0.89	–
11	*100*	*89.5* ± *1.22*	*100*	10.5 ± 1.13	–	44.2 ± 1.11	*100*	19.2 ± 0.57
13	3.5 ± 0.55	11.5 ± 1.19	47.2 ± 0.63	1.3 ± 0.78	*100*	74.3 ± 0.52	35.8 ± 0.91	84.2 ± 1.61
14	38.5 ± 1.41	5.6 ± 0.47	45.3 ± 1.28	2.3 ± 0.66	65.3 ± 1.44	*100*	47.2 ± 1.47	–
15	56.5 ± 0.56	49.6 ± 1.23	*91.5* ± *1.74*	–	–	*100*	*94.5* ± *0.54*	–
16	35.9 ± 0.70	5.9 ± 0.78	44.6 ± 0.25	40.3 ± 1.22	34.2 ± 0.13	55.3 ± 0.87	53.2 ± 1.33	–
17	7.3 ± 0.35	7.5 ± 0.92	51.6 ± 1.18	3.1 ± 1.14	71.2 ± 1.45	59.3 ± 0.68	56.3 ± 0.96	–
DOX	100	100	100	100	100	100	100	100
Negative control	0	0	0	0	0	0	0	0

Significant values are in italics.

#### Secondary screening

As depicted in Table 6, the most promising compounds against HCT116 were compounds **8** and **9** with IC_50_ values 47.3 and 17.8 µM, respectively compared to doxorubicin (69.2 µM). Regarding HePG2 cell line, compound **9** showed the highest activity with an IC_50_ value 12.4 µM as compared to doxorubicin (39.7 µM), while compounds **7** and **8** exerted moderate activity with IC_50_ values of 97.4 and 74 µM respectively. However, compound **8** exerted lower activity than compound **9** against HCT116 and HePG2, it was more selective. Compounds **7** and **8** exhibited promising activity against A549 and PACA2 with IC_50_ values 86.5 and 44.4 µM respectively for compound **7**, and 55.8 and 22.4 µM respectively for compound **8**. While compounds **11** and **15** showed moderate activity compared to doxorubicin (IC_50_ = 52.1 µM) against PACA2 with IC_50_ values 80.7 and 82.5 µM respectively. Regarding PC3, compound **8** was the most active one with an IC_50_ value of 57.2 µM, while compounds **7** and **11** showed moderate activity with IC_50_ values of 91.3 and 114.7 µM respectively. The most active compound against HOS was compound **9** with an IC_50_ value of 17.6 µM, also compound **15** showed promising activity with an IC_50_ value of 37.3 µM, while compound **7** showed mild activity with an IC_50_ value of 102.15 µM compared to doxorubicin (IC_50_ = 24.3 µM). Regarding A431, only compound **9** exerted activity which was moderate with an IC_50_ value of 113.3 µM. From these results, it was noticed that compounds **7**, **8**, **9** and **15** exerted the best cytotoxic effect and compound **8** was the most selective one. So, further molecular studies were done on these selected compounds.

**Table 6:** IC_50_ (µM) of the compounds **7–11** and** 13–17**.

Comp	A549	HCT116	PC3	A431	HePG2	HOS	PACA2	BJ1
7	86.5 ± 1.40	93.5 ± 1.40	91.3 ± 0.28	–	97.4 ± 1.11	102.1 ± 0.95	*44.4* ± *0.57*	48.8 ± 0.26
8	*55.8* ± *0.11*	*47.3* ± *0.91*	*57.2* ± *0.39*	–	74.0 ± 0.29	–	*22.4* ± *0.49*	–
9	–	*17.8* ± *0.35*	–	113.3 ± 0.68	*12.4* ± *0.17*	*17.6* ± *0.24*	–	52.5 ± 0.74
10	–	–	–	–	–	–	–	–
11	149.3 ± 0.23	161.6 ± 0.24	114.7 ± 0.21	–	–	–	86.7 ± 0.18	–
13	–	–	–	–	121.7 ± 0.85	–	–	–
14	–	–	–	–	–	*61.6* ± *0.81*	–	–
15	–	–	–	–	–	*37.3* ± *0.37*	82.5 ± 0.21	94.4 ± 0.71
16	–	–	–	–	–	–	–	–
17	–	–	–	–	199.3 ± 0.82	–	–	–
DOX	52.1 ± 0.28	69.2 ± 0.10	43.8 ± 0.47	45.8 ± 0.15	39.7 ± 0.18	24.3 ± 0.24	52.1 ± 0.19	24.8 ± 0.52

Significant values are in italics.

### Gene expression analysis

#### Gene expression in pancreatic cell line

**Figure 4:**
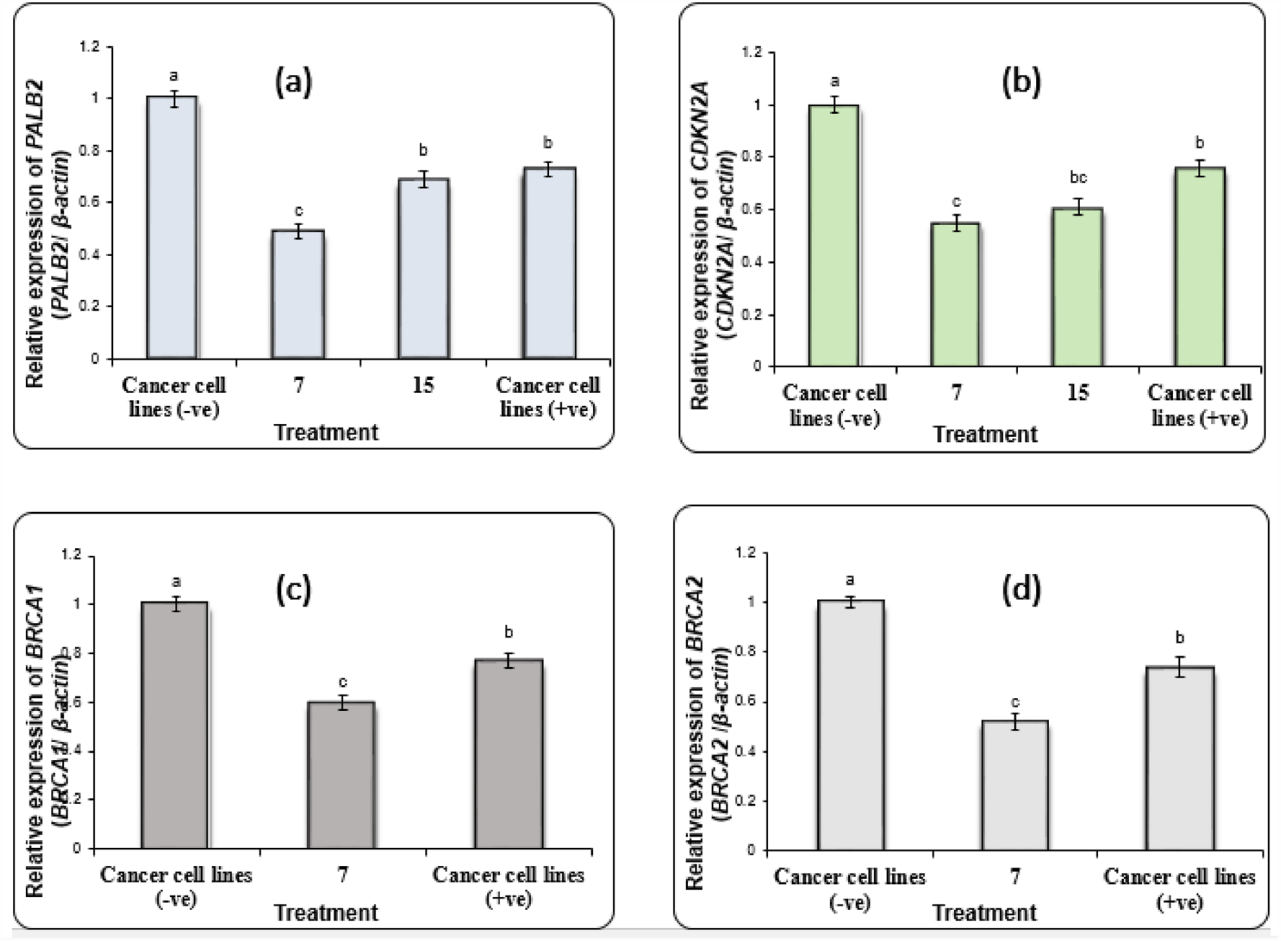
Alterations in the gene expression level of (**a**)* PALB2* and (**b**) CDKN2A; genes in Paca2 cancer cell line treated with **7** and **15**. (**c**) *BRCA1* and (**d**) *BRCA2*; genes in PC3 cancer cell lines treated with **7**. Data are presented as mean ± SEM. (**a**, **b**, **c**) Mean values within tissue with unlike superscript letters were significantly different (P < 0.05).

The gene expression result of selected gene *PALB2* (Partner And Localizer Of BRCA2) and *CDKN2A* in PACA2 cells revealed that the treated pancreatic cell line showed a significant reduction (P < 0.01) in the expression levels of *PALB2* and *CDKN2A* genes compared with negative untreated sample (-ve) (Fig. 4a,b). Also, compared to the negative sample of the pancreatic cancer cell line, the expression values of *PALB2* and *CDKN2A* genes were significantly reduced (P < 0.05) in the treated (**7** and **15**) and positive control pancreatic cell line (doxorubicin). Moreover, the *PALB2* gene expression level was significantly suppressed in treated **7** and **15** versus positive control PACA2 cells (+ ve). *PALB2* gene plays an important role in double-strand break repair and its down-regulation resulted in DNA damage in the treated cells^47^. This was confirmed in the subsequent sections of the DNA damage analysis using comet and gel electrophoresis assays.

#### Gene expression in prostate cancer cell line

The *BRCA1* (Breast cancer 1) and *BRCA2* (Breast cancer 2) genes, which are associated with PC3 prostatic carcinoma, were used to perform gene expression analysis in these tissue. The results presented in Figs. 4c & d indicated that negative samples of PC3 showed significantly high expression levels of *BRCA1* and *BRCA2* genes (P < 0.01) compared with treated PC3 cells. While the expression values of *BRCA1* and *BRCA2* genes were decreased significantly (P < 0.05) in treated **7** and positive control PC3 cells compared with negative samples. Additionally, the *BRCA1* and *BRCA2* gene expression levels were downregulated in **7** much lower than those in positive control PC3 cells (doxorubicin). *BRCA1* and *BRCA2* genes are implicated in DNA repair and hence decreasing the expression level of them leads to DNA fragmentation^48^. Herein, the downregulation of *BRCA1* and *BRCA2* supported our results in the subsequent DNA damage sections.

#### Gene expression in lung cancer cell line

**Figure 5:**
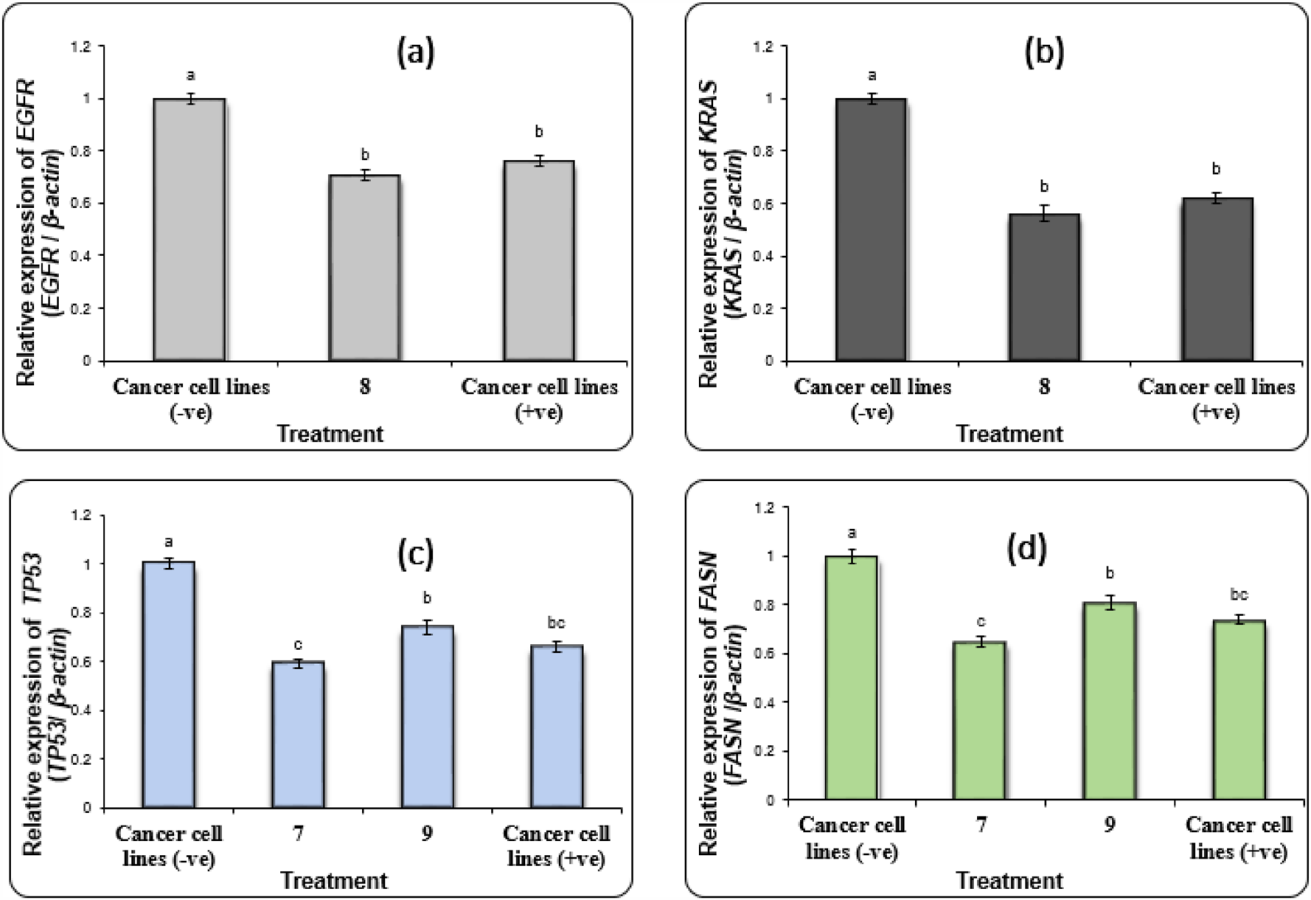
Alterations in the gene expression of (**a**) *EGFR* and (**b**) *KRAS*; genes in A549 cancer cell lines. (**c**) *TP53* and (**d**) *FASN*; genes in HCT116 cancer cells treated with **7** and **9**. Data are presented as mean ± SEM. (**a**, **b**, **c**) Mean values within tissue with unlike superscript letters were significantly different (P < 0.05).

The results of selected genes *EGFR* (Epidermal Growth Factor Receptor) and *KRAS* (Kirsten rat sarcoma virus) in A549 lung cancer cell line showed a significant increase of their expression levels (P < 0.01) in negative samples of A549 cells compared with treated samples (Fig. 5a,b). While the treated **8** and doxorubicin-treated A549 cells showed a significant reduction of *EGFR* and *KRAS* gene expression levels (P < 0.05) compared with negative samples. Likewise, the expression levels of *EGFR* and *KRAS* genes were downregulated in treated **8** much lower than those in positive control A549 cells. In lung carcinoma, *EGFR* and *KRAS* are found to be tumorigenesis drivers and are implicated in invasion and metastasis processes^49,50^. So, compound **8** exerted anticancer activity through the down-regulation of *EGFR* and *KRAS* in A549 lung carcinoma.

#### Gene expression in colon cancer cell line

The results of *TP53* (tumor protein p53) and *FASN* (Fatty Acid Synthase) genes expression analysis in HCT116 cells revealed that the expression levels of *TP53* and *FASN* genes were improved significantly (P < 0.01) in negative samples compared with treated samples of HCT116 cells (Fig. 5c,d). Where a suppression (P < 0.05) in the expression levels of *TP53* and *FASN* genes was recorded in **7**, **9** and doxorubicin (positive control) treated HCT116 cells compared with negative samples. Also, **7** and doxorubicin-treated HCT116 cells showed a significant decrease in *TP53* and *FASN* gene expression levels compared with **9** treated cells. The lipid metabolism provides an alternative source of energy required to afford tumor growth. FASN plays a central role in lipid metabolism and hence helps in the survival of tumor cells^51^. So, compounds **7** and **9** had an antisurvival effect on tumor cells via decreasing the expression level of the FASN gene.

### DNA damage analysis

#### DNA damage in pancreatic cell line

**Figure 6:**
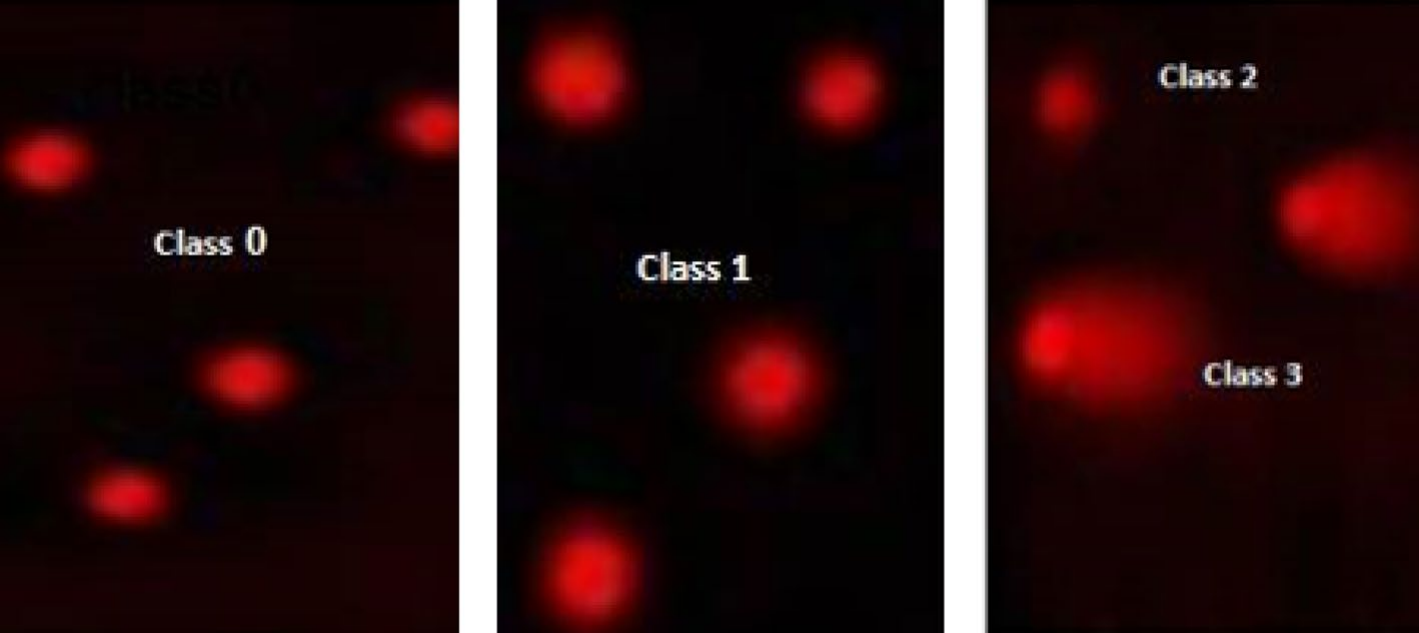
The visual score of normal DNA (class 0) and damaged DNA (classes 1, 2 and 3) using the comet assay.

The data in Table 7 and Fig. 6 represented the DNA damage in the PACA2 cell line. The mean values of DNA damage were significantly decreased (P < 0.05) in negative untreated samples of the PACA2 cell line compared with treated cell lines. On the other hand, the DNA damage values were increased significantly (P < 0.01) in treated **7** and **15** and doxorubicin-treated cells and the highest values of DNA damage were recorded in **7** much more than those in **15** and doxorubicin-treated cells.

**Table 7:** Visual score of DNA damage in **7** and **15** treated PACA2, **7** treated PC3, **8** treated A549, and **7** and **9** treated HCT116. Doxorubicin was used as a positive control.

Treatment	Cell line	No. of samples	No. of cells		Class**				DNA damaged cells % (mean ± SEM)
			Analyzed*	Comets	0	1	2	3	
Untreated cells	PACA2	4	400	43	357	31	10	2	10.76 ± 1.11^b^
7		4	400	94	306	35	28	31	23.52 ± 0.65^a^
15		4	400	84	316	31	25	28	21.00 ± 1.29^a^
Doxorubicin		4	400	81	319	28	26	27	20.25 ± 0.75^a^
Untreated cells	PC3	4	400	41	359	29	11	1	10.27 ± 0.85^c^
7		4	400	103	297	38	36	29	25.78 ± 1.44^a^
Doxorubicin		4	400	83	317	39	30	14	20.76 ± 1.38^b^
Untreated cells	A549	4	400	43	357	36	7	0	10.79 ± 0.48^b^
8		4	400	99	301	39	38	22	24.75 ± 1.03^a^
Doxorubicin		4	400	86	314	25	32	29	21.52 ± 1.04^a^
Untreated cells	HCT116	4	400	42	358	31	8	3	10.53 ± 1.19^c^
7		4	400	102	298	29	38	35	25.56 ± 0.66^a^
9		4	400	79	321	37	24	18	19.75 ± 1.25^b^
Doxorubicin		4	400	85	315	33	24	28	21.26 ± 0.48^ab^

*Number of cells examined per a group, **Class 0 = no tail; 1 = tail length < diameter of nucleus; 2 = tail length between 1 and 2 × the diameter of nucleus; and 3 = tail length > 2 × the diameter of nucleus. ^a–c^Mean values within tissue with unlike superscript letters were significantly different (P < 0.05).

#### DNA damage in prostate cell line

As shown in Table 7 and Fig. 6, the DNA damage values in the PC3 cancer cell line were significantly decreased (P < 0.05) in negative untreated samples of the PC3 compared with the treated cells. Meanwhile, the treated **7** and doxorubicin-treated PC3 cells showed a significant increase (P < 0.01) in DNA damage values whereas the highest values were observed in **7** much more than those in doxorubicin-treated cells.

#### DNA damage in lung cell line

The results of DNA damage in the A549 cancer cell line showed that negative untreated samples of the A549 exhibited a significant decrease (P < 0.05) in DNA damage values compared with treated cell lines (Table 7 and Fig. 6). However, the DNA damage values were increased significantly (P < 0.01) in **8** and doxorubicin-treated A549 samples and the highest values of DNA damage were observed in **8** treated cells much more than those in the doxorubicin-treated sample.

#### DNA damage in the colon cell line

The current results showed a significant decrease (P < 0.05) in DNA damage values in negative untreated samples of HCT116 compared with treated (Table 7 and Fig. 6). Further, treated **7** and doxorubicin HCT116 cells showed a significant increase (P < 0.01) in DNA damage values and the highest values were observed in **7** much more than those in doxorubicin and **9** treated cells.

### DNA fragmentation analysis

#### Measurement of DNA fragmentation in pancreatic cancer cell line

The effect of different treatments on DNA damage in PACA2 cancer cell line revealed that DNA fragmentation rates were significantly increased in treated samples **7** and **15** and doxorubicin compared with negative untreated samples as summarized in Table 8 and Fig. 4. However, the DNA fragmentation values were increased significantly (P < 0.01) in treated PACA2 samples compared with negative control. Whereas, the highest value of DNA fragmentation was observed in **7** much more than **15** and those in doxorubicin-treated PACA2 cells.

**Table 8:** DNA fragmentation results were detected in PACA2 treated with **7** and **15,** PC3 treated with **7,** A549 treated with **8,** and HCT116 treated with **7** and **9**.

Treatment	Cell line	DNA fragmentation % M ± SEM	Change	Inhibition
Untreated cells	PACA2	12.1 ± 0.59^c^	0.00	0.0
7		35.2 ± 0.68^a^	23.10	28.33
15		32.3 ± 0.74^ab^	20.20	12.22
Doxorubicin		30.1 ± 0.62^b^	18.00	0.0
Untreated cells	PC3	11.6 ± 0.47^c^	0.0	0.0
7		36.1 ± 0.60^a^	24.5	21.89
Doxorubicin		31.7 ± 0.85^b^	20.1	0.0
Untreated cells	A549	11.4 ± 0.38^b^	0.0	0.0
8		34.2 ± 0.57^a^	22.8	16.92
Doxorubicin		30.9 ± 1.64^a^	19.5	0.0
Untreated cells	HCT116	10.9 ± 0.65^b^	0.00	0.00
7		37.1 ± 0.52^a^	26.20	24.76
9		29.0 ± 0.54^a^	18.10	-13.81
Doxorubicin		31.9 ± 0.82^a^	21.00	0.00

Means with different superscripts (a, b, c) between treatments in the same column are significantly different at *P* < 0.05.

#### Measurement of DNA fragmentation in prostate cancer cell line

The data in Table 8 and Fig. 4 revealed that the rate of DNA fragmentation in PC3 cancer cell line was significantly suppressed (P < 0.01) in negative samples of the PC3 compared with those in treated samples **7** and doxorubicin (positive control). Although, the DNA fragmentation values were increased significantly (P < 0.01) in treated PC3 samples compared with negative control, the highest values were found in **7** treated cells as compared to the doxorubicin-treated cells.

#### Measurement of DNA fragmentation in lung cancer cell line

**Figure 7:**
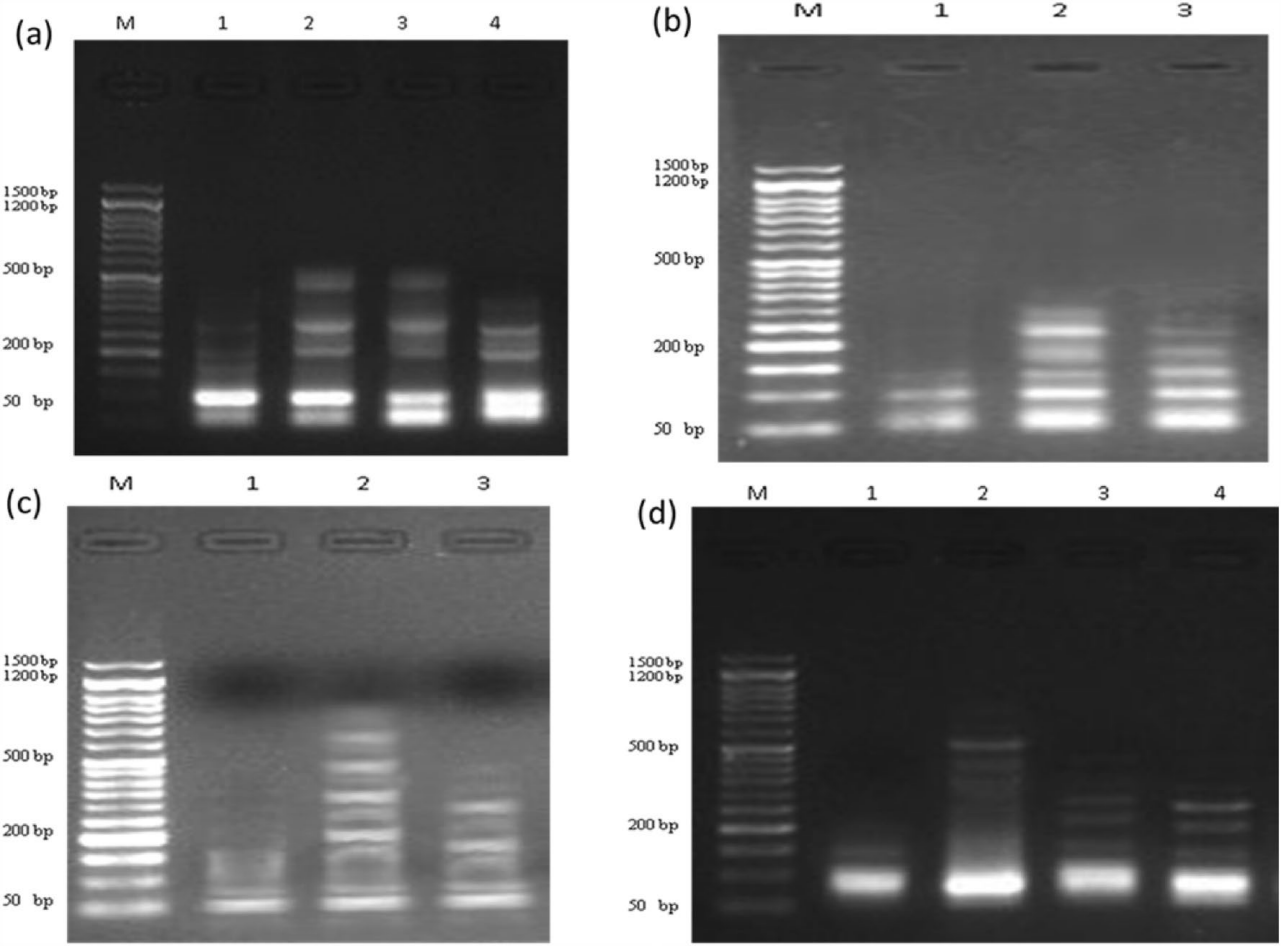
DNA fragmentation detected with Agarose gel in (**a**) PACA2 (lane 1: negative control, lane 2: **7**, lane3: **15**, lane 4: positive control); (**b**) PC3 (lane 1: negative control, lane 2: **7**, lane 3: positive control); (**c**) A549 (lane 1: negative control, lane 2: **8**, lane 3: positive control); (**d**) HCT116 (lane 1: negative control, lane 2: **7**, lane 3: **9**, lane 4: positive control), M: represent DNA marker.

The results of DNA fragmentation in A549 determination as presented in Table 8 and Fig. 7 showed that negative samples of A549 exhibited a significant decrease (P < 0.01) in DNA fragmentation rates compared with those in **8** and doxorubicin-treated samples. However, treated A549 samples showed a significant reduction in the DNA fragmentation values (P < 0.01) compared with negative control. Moreover, the highest value of DNA fragmentation was observed in **8** much more than those in doxorubicin-treated cells.

#### Measurement of DNA fragmentation in colon cancer cell line

The results of DNA fragmentation in HCT116 revealed that negative samples HCT116 showed a significant decrease (P < 0.01) in DNA fragmentation rates compared with those in treated samples **7**, **9** and doxorubicin (positive control) (Table 8 and Fig. 7). However, the DNA fragmentation values were increased significantly (P < 0.01) in treated HCT116 samples compared with negative control. Additionally, the highest value of DNA fragmentation was observed in **7** much more than those in doxorubicin and **9** treated samples. DNA fragmentation into oligonucleosomal fragments is a biochemical feature of programmed cell death (apoptosis)^52^. It is worth to be mentioned that DNA fragmentation is a late event of the apoptosis process. So, our prepared evaluated compounds induced apoptosis in the above-mentioned cancer cell lines.

#### Molecular docking study

**Figure 8:**
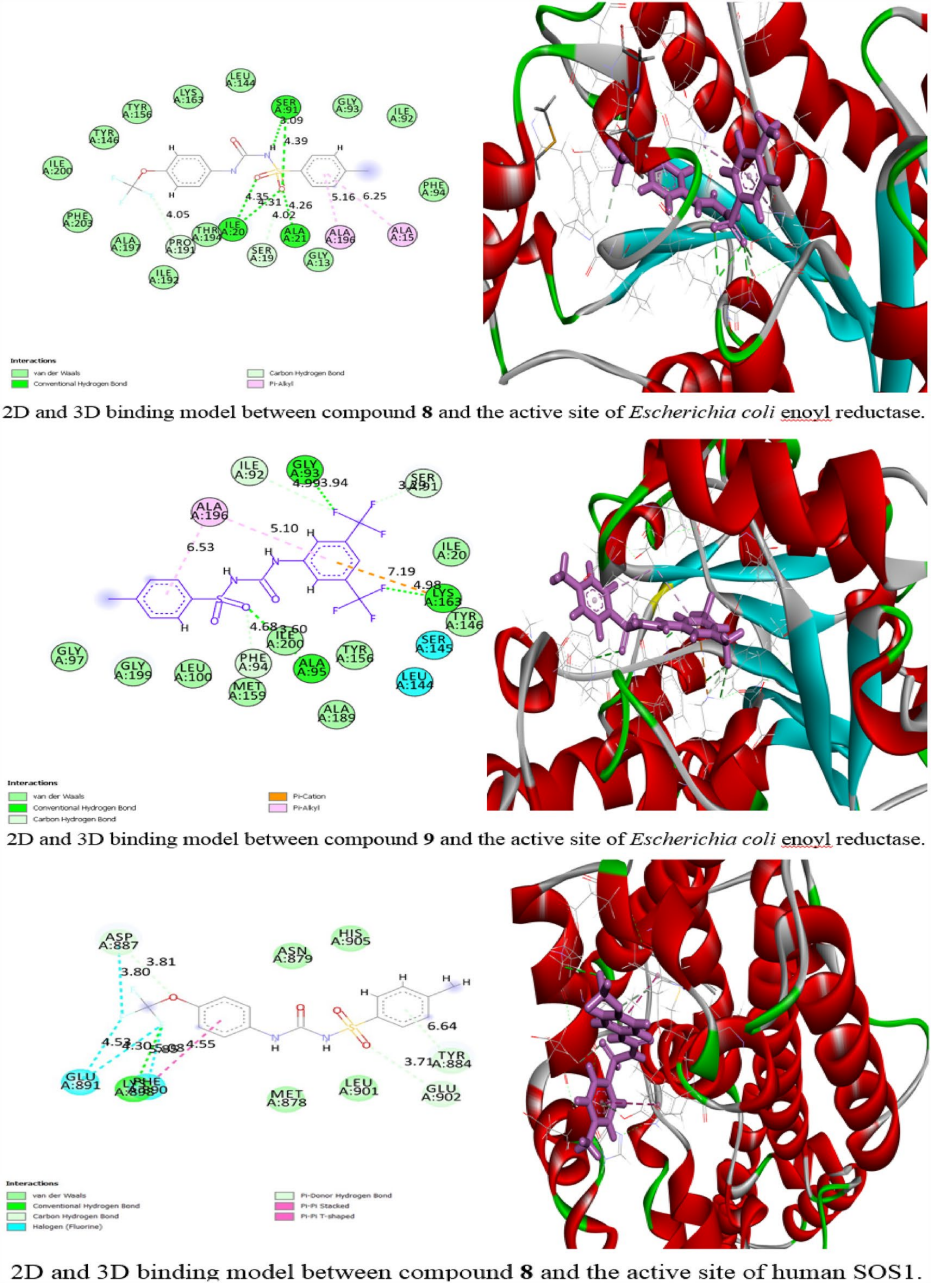
The molecular interactions of compounds **8** and **9** with the active site of *E. coli* enoyl reductase and compound **8** with the active site of human SOS1.

Compounds **8** and** 9** were chosen for the molecular docking study. As it was seen from the anticancer activity section, compounds **8** and** 9** had the best cytotoxic effect against the majority of the evaluated cancer cell lines and had a little cytotoxic effect on the evaluated normal cell line. Also, they had promising antimicrobial activity. Compounds **8** and **9** were studied against *Escherichia coli* enoyl reductase and only compound **8** was studied against human Son of sevenless homolog 1 (SOS1). The proteins were downloaded from the protein database with their co-crystalized ligands which were used as a reference standard ligand. It was found that the root mean squared deviation (RMSD) of the co-crystalized ligand were (1.34, and 0.5 A^0^) for *Escherichia coli* enoyl reductase and SOS1, respectively. It was noticed that the RMSD value was ≤ 2 which confirmed the accuracy of the docking parameters. Regarding the bacterial protein (*E. coli* enoyl reductase), the binding energy (S) were − 23.1 and − 18.9 kcal/mol for compounds **8** and** 9** respectively which were better than that of the standard (− 16.8 kcal/mol) (Table 9). While, for the human protein (SOS1), the S value of compound **8** was equaled − 18.7 kcal/mol. As compared to the S value of the standard ligand (− 23.8 kcal/mol) (Table 9), compound **8** showed lower binding energy but was still in negative charge which demonstrated the spontaneous interaction with the selected protein. As shown in Fig. 8, compound **8** interacted with the active site of *E. coli* enoyl reductase through seven hydrogen bonds, one between fluorine atom and PRO 191 with bond distance 4.05 A°, two hydrogen bonds between the amino acid SER 91and the oxygen of the SO_2_ and NH of amide group with bond distances 4.39 and 3.09 A° respectively. Two hydrogen bonds between oxygen of SO_2_ and two different amino acids, ALA 21 and SER 19 with bond distances 4.26 and 4.02 A° respectively. The last two hydrogen bonds were between ILE 20 and two different oxygens of the SO_2_ group with comparable bond distances of 4.31 and 4.35 A°. Also, there were two electrostatic interactions between the benzene ring, ALA 15 and ALA 196. Regarding compound **9**, there were eight interactions with the *E. coli* enoyl reductase enzyme (Fig. 8). These interactions included, two hydrogen bonds between a fluorine atom and both GLY 93 and ILE 92 with bond distances of 3.9 and 4.9 A° respectively, a hydrogen bond between another fluorine atom and LYS 163 with a bond distance 4.98 A°, two hydrogen bonds between the oxygen of the SO_2_, ILE 200 and PHE 94 with bond distances 3.6 and 4.68 A° respectively, two electrostatic interactions between two different benzene rings and ALA 196, a third electrostatic interaction was seen between benzene and LYS 163. Regarding human SOS1 protein, it was found that compound **8** interacted through nine interactions; four interactions between fluorine atoms and PHE 890, ASP 887, and GLU 891 residues, one conventional hydrogen bond which was between fluorine atom and LYS 898 with bond distance 5.8 A°, pi-donor hydrogen bond between benzene ring and TYR 884 with bond distance 6.64 A°, another two hydrogen bonds with GLU 902 and ASP 887 with bond distances 3.7 and 3.8 A° respectively and pi-pi stacked between benzene ring and PHE 890 (Fig. 8). Enoyl reductase enzyme is responsible for the last step of the fatty acid biosynthesis in *E.coli* bacteria^53^. According to the molecular docking study, the antibacterial activity of our prepared compounds might be due to the inhibition of the enoyl reductase enzyme. The human SOS1 is a co-activator transcriptional factor for the *KRAS* gene^54^. The inhibition of the SOS1 protein leads to the down-regulation of the *KRAS* gene. Our result in the above-mentioned gene expression analysis of* KRAS* in A549 lung carcinoma confirmed our result of the molecular docking study on SOS1 protein.

**Table 9:** The Gibbs free energy (kcal/mole) between the selected proteins and the compounds **8** and **9**. The co-crystalized ligand was used as a standard.

Compound	*E. coli* enoyl reductase	Compound	Son of sevenless homolog 1
**8**	− 23.1	**8**	− 18.7
**9**	− 18.9		
Co-crystallized ligand	− 16.8	Co-crystallized ligand	− 23.8

#### Structure–activity relationship (SAR)

**Figure 9:**
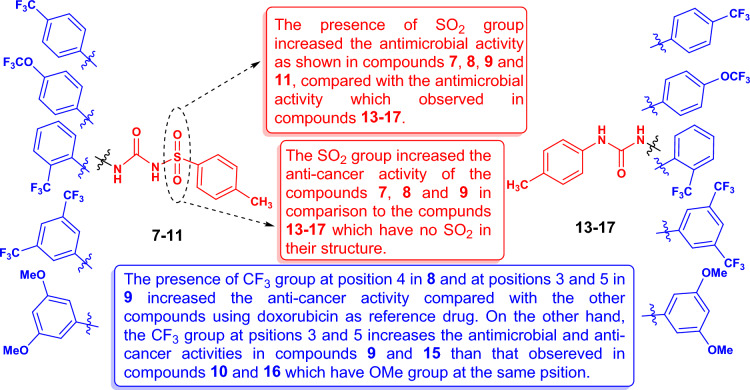
SAR of the newly synthesized urea compounds **7–11** and** 13–17**.

To explain the relationship between the chemical structure and the biological properties of the newly synthesized compounds, the Structure–Activity Relationship (SAR) of urea derivatives **7–11** and **13–17** was visualized in Fig. 9. The presence of the sulfonyl group in compounds **7–11** is essential for increasing the antimicrobial and anti-cancer activity of these compounds in comparison with compounds **13–17** as the non-containing sulfonyl group. Compounds **7**, **8**, **9**, and **11** were found as the most antibacterial agents compared to all the examined compounds, with the most potent effect on both *Bacillus mycoides* (36, 37, 41, and 31 mm, respectively) and *Candida albicans* (37, 38, 41, and 32 mm, respectively), while the minimum inhibitory concentrations (MICs) of these compounds were determined against *B. mycoides*, *E. coli*, and *C. albicans*, and compound **8** is the most active at 4.88 µg/mL. On the other hand, compounds **7**, **8** and **9** (with sulfonyl group) showed potent anti-cancer properties against most of the tested cell lines. For example, compound **8** has strong anti-cancer activity against A549, HCT116, PC3 and PACA2 cell lines with IC_50_ 55.8, 47.3, 57.2 and 22.4 µM, respectively, and compound **9** gave excellent anti-cancer activity against HCT116, HePG2 and HOS cell lines with IC_50_ 17.8, 12.4 and 17.6 µM, respectively. The presence of the trifluoromethyl group at positions 3 and 5 in compounds **9** and **15** increased the anti-cancer properties than that observed in compounds **10** and **16** which have methoxy groups at positions 3 and 5. It was worth noting that, the presence of the trifluoromethoxy group at position 4 in compound **14** increased the anti-cancer activity on the HOS cell line with IC_50_ 61.6 µM even in the absence of the sulfonyl group. Likewise, the presence of trifluoromethyl group at positions 3 and 5 increased the anti-cancer properties on HOS cell line in the presence of sulfonyl group (as in compound **9** with IC_50_ 17.6 µM) or absence of sulfonyl group (as in compound **15** with IC_50_ 37.3 µM).

## Conclusion

A novel series of sulfonylurea derivatives (**7–11**), and aryl-urea derivatives (**13**–**17**) were evaluated as antimicrobial and anticancer agents. The current study's antimicrobial results demonstrated the efficacy of the synthesized derivatives as antimicrobial agents versus diverse microbial populations. That data can aid in the development of innovative medications to combat the challenge of resistant pathogens. As anticancer candidates, compounds **7**, **8**, **9** and **15** showed promising activity with the lowest IC_50_ values as compared to doxorubicin against different cancer cell lines. The anticancer activity of these promising compounds was studied at the gene expression level which showed the down-regulation of *PALB2*, (*BRCA1* and *BRCA2*), (*EGFR* and *KRAS*) and (*TP53* and *FASN*) in PACA2, PC3, A549 and HCT116 treated cells respectively. It was concluded from the percentage of DNA damage that was studied via two different techniques that our evaluated compounds induced apoptotic cell death in the treated cancer cell lines. The molecular docking study revealed the inhibition possibility of compounds **8** and **9** against the *E. coli* enoyl reductase and compound **8** against human SOS1. The structure–activity relationship revealed the great impact of the added sulfonyl group on the activity of our prepared urea derivatives.

### Supplementary Information

Supplementary Figures.

## Data Availability

The data that support the findings of this study are available from the corresponding author upon reasonable request.
