# Supplementary material for: Impact of trifluoromethyl and sulfonyl groups on the biological activity of novel aryl-urea derivatives: synthesis, in-vitro, in-silico and SAR studies
Source: Sci Rep. 2023 Oct 16;13:17560. doi: 10.1038/s41598-023-44753-9 (PMC10579241; doi:10.1038/s41598-023-44753-9)
Supplement: Supplementary file 1 — Supplementary Figures. [file 41598_2023_44753_MOESM1_ESM.docx]

Electronic Supporting Information for

**Impact of trifluoromethyl and sulfonyl groups on the on the antimicrobial and anti-cancer activities of novel aryl-urea derivatives: Synthesis, *in-vitro*, *in-silico* and SAR studies**

Farid M. Sroor ^1,^ *, Karima F. Mahrous^2^, Heba A. M. Abd El-Kader^2^, Abdelmageed M. Othman^3^, Nada S. Ibrahim^4^

1. Organometallic and Organometalloid Chemistry Department, National Research Centre, 12622 Cairo, Egypt
2. Cell Biology Department, National Research Centre, 12622 Dokki, Egypt
3. Microbial Chemistry Department, Biotechnology Research Institute, National Research Centre, 12622 Dokki, Egypt
4. Department of Chemistry (Biochemistry Branch), Faculty of Science, Cairo University, Giza, Egypt

* Corresponding author

*Email address*: [faridsroor@gmx.de](mailto:faridsroor@gmx.de), [fm.sroor@nrc.sci.eg](mailto:fm.sroor@nrc.sci.eg)

***Contents***

| ^1^H and ^13^C-NMR spectra of **7** | S2 & S3 |
| --- | --- |
| ^1^H and ^13^C-NMR spectra of **8** | S4 & S5 |
| ^1^H and ^13^C-NMR spectra of **9** | S6 & S7 |
| ^1^H and ^13^C-NMR spectra of **10** | S8 & S9 |
| ^1^H and ^13^C-NMR spectra of **11** | S10 & S11 |
| ^1^H and ^13^C-NMR spectra of **13** | S12 & S12 |
| ^1^H and ^13^C-NMR spectra of **14** | S14 & S15 |
| ^1^H and ^13^C-NMR spectra of **15** | S16 & S17 |
| ^1^H and ^13^C-NMR spectra of **16** | S18 & S18 |
| ^1^H and ^13^C-NMR spectra of **17** | S20 & S21 |


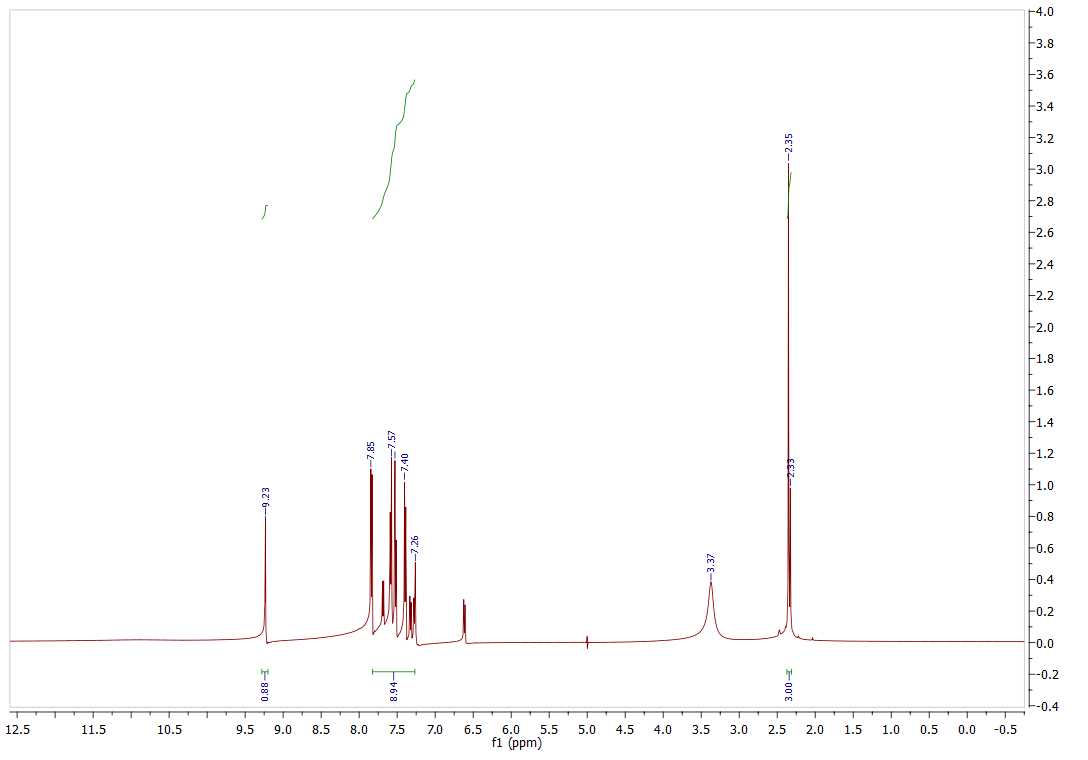


^1^H-NMR spectra of **7**


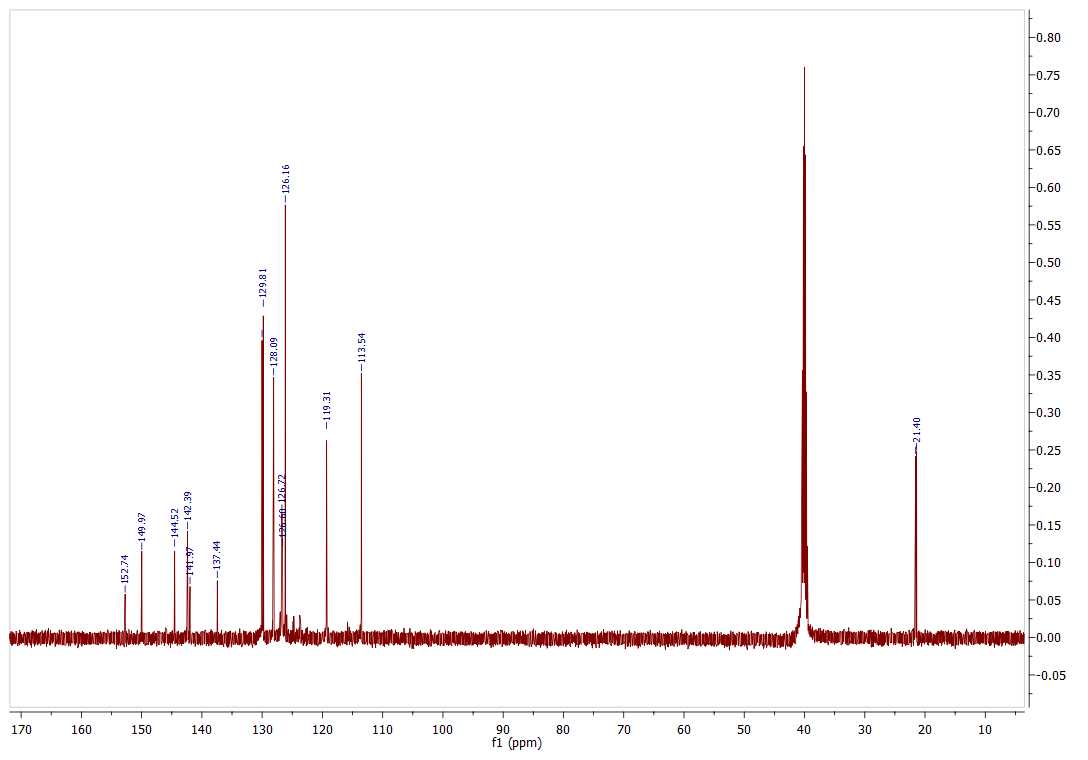


^13^C-NMR spectra of **7**

**
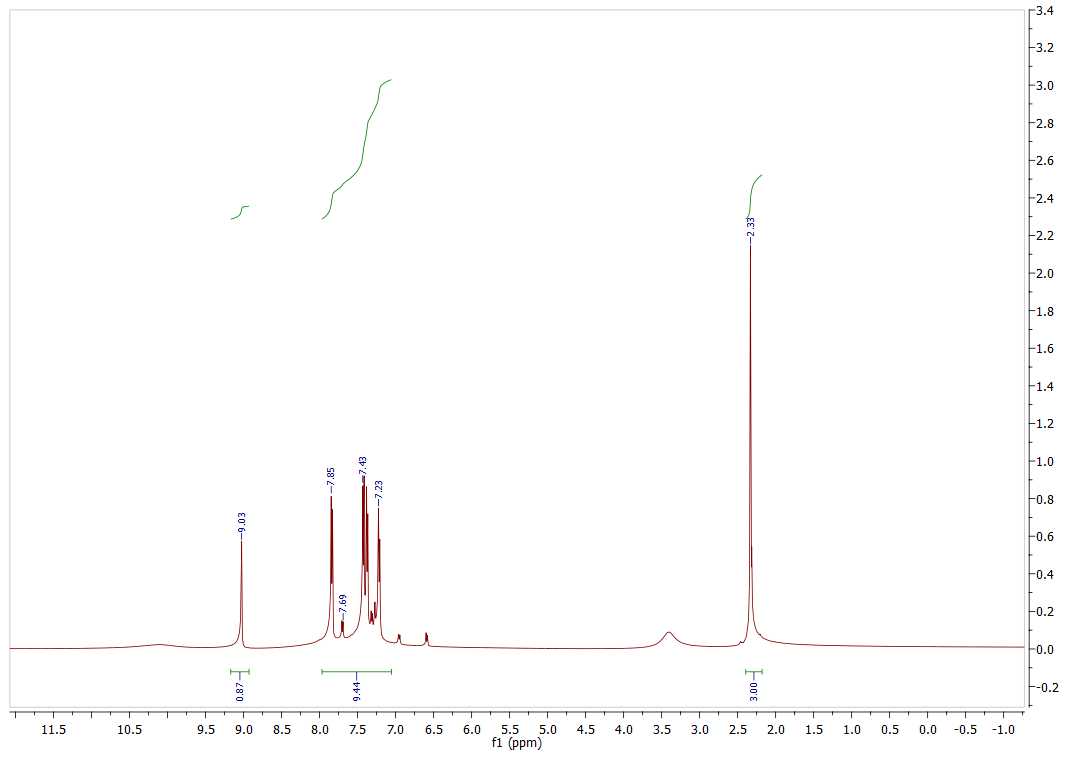
**

^1^H-NMR spectra of **8**


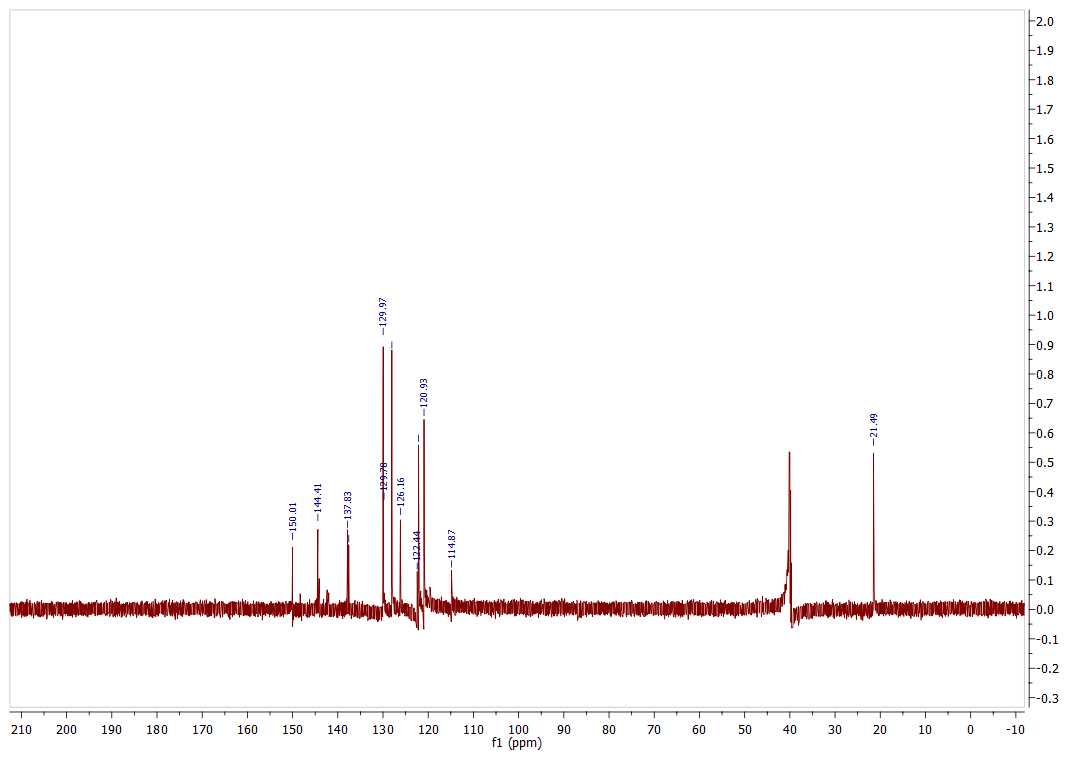


^13^C-NMR spectra of **8**


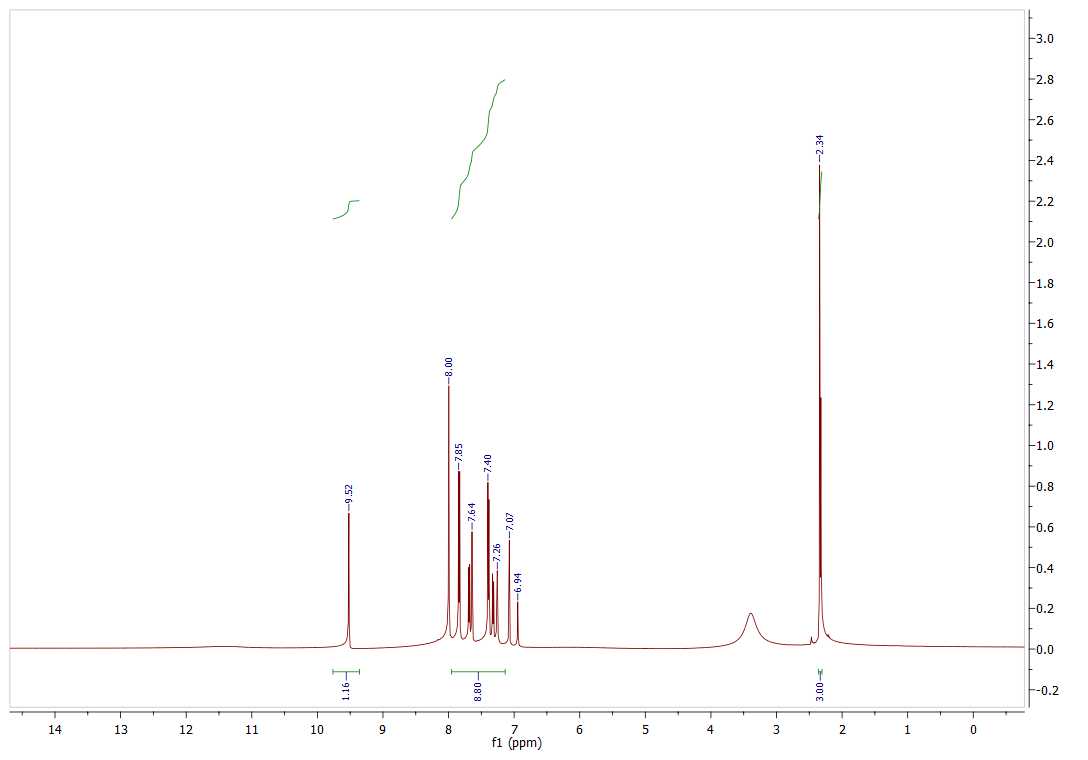


^1^H-NMR spectra of **9**


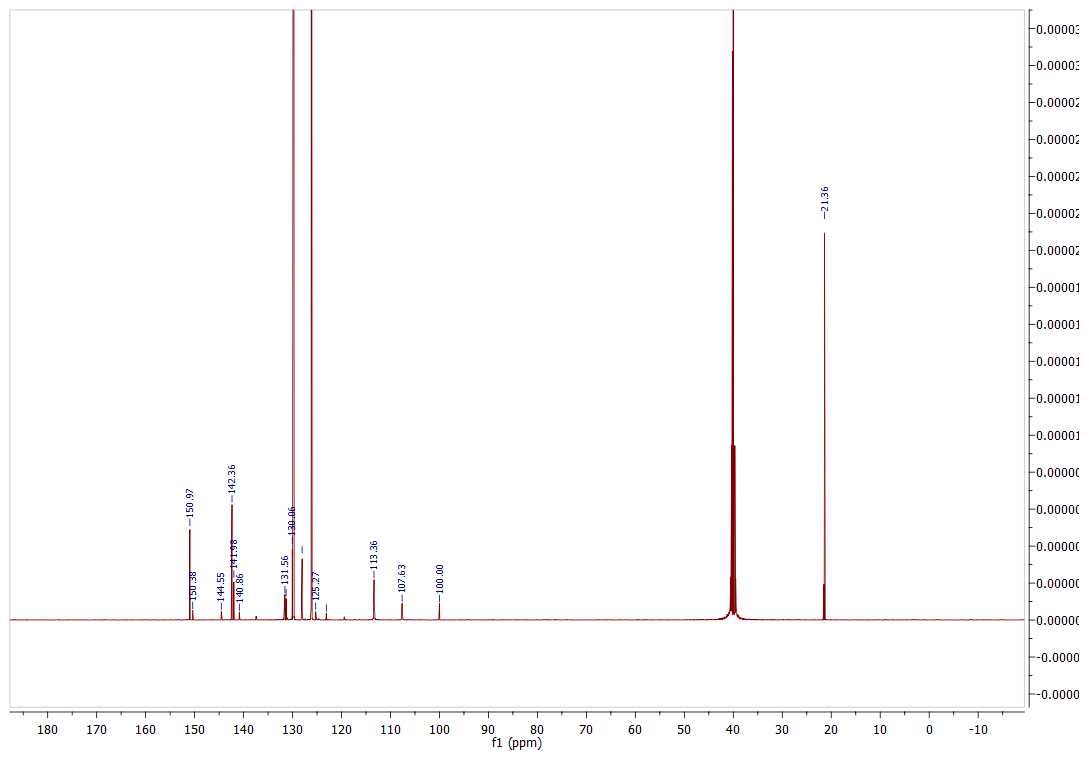


^13^C-NMR spectra of **9**

^
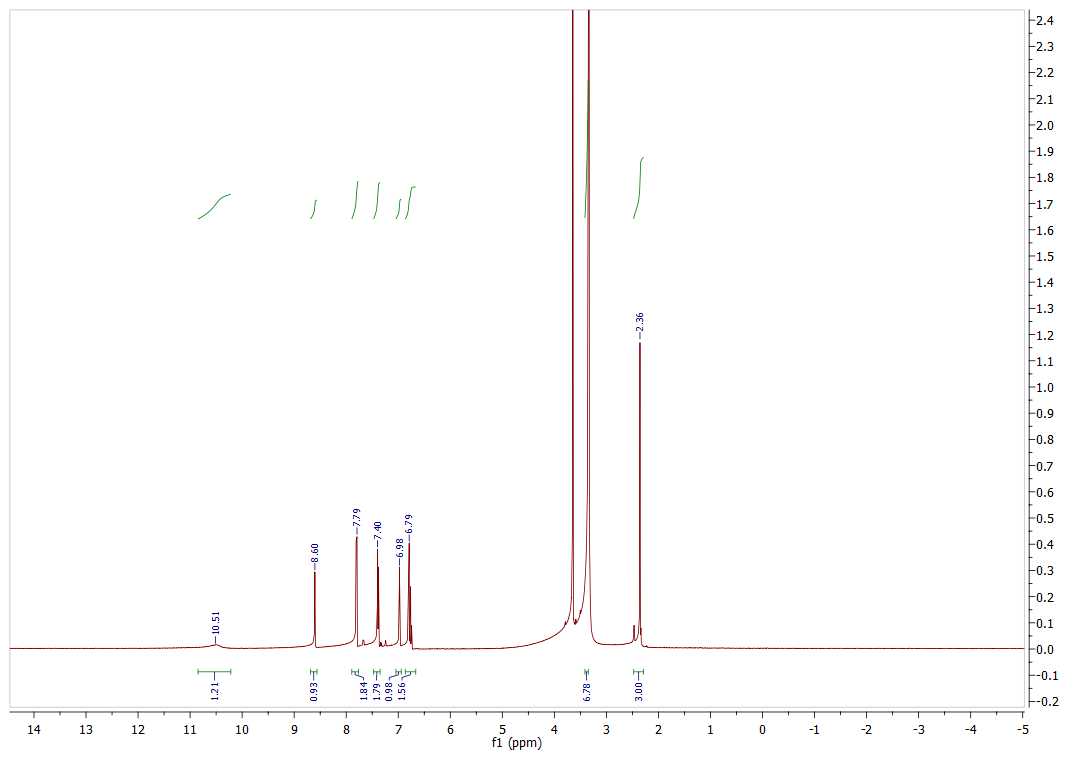
^

^1^H-NMR spectra of **10**


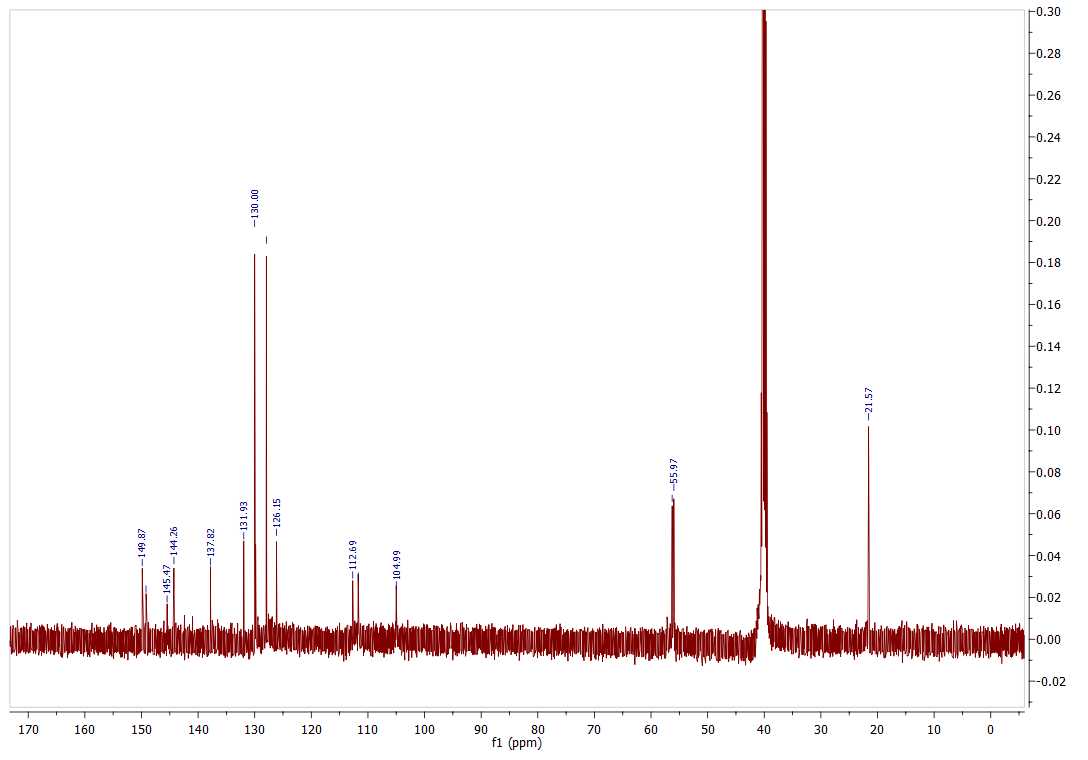


^13^C-NMR spectra of **10**

^
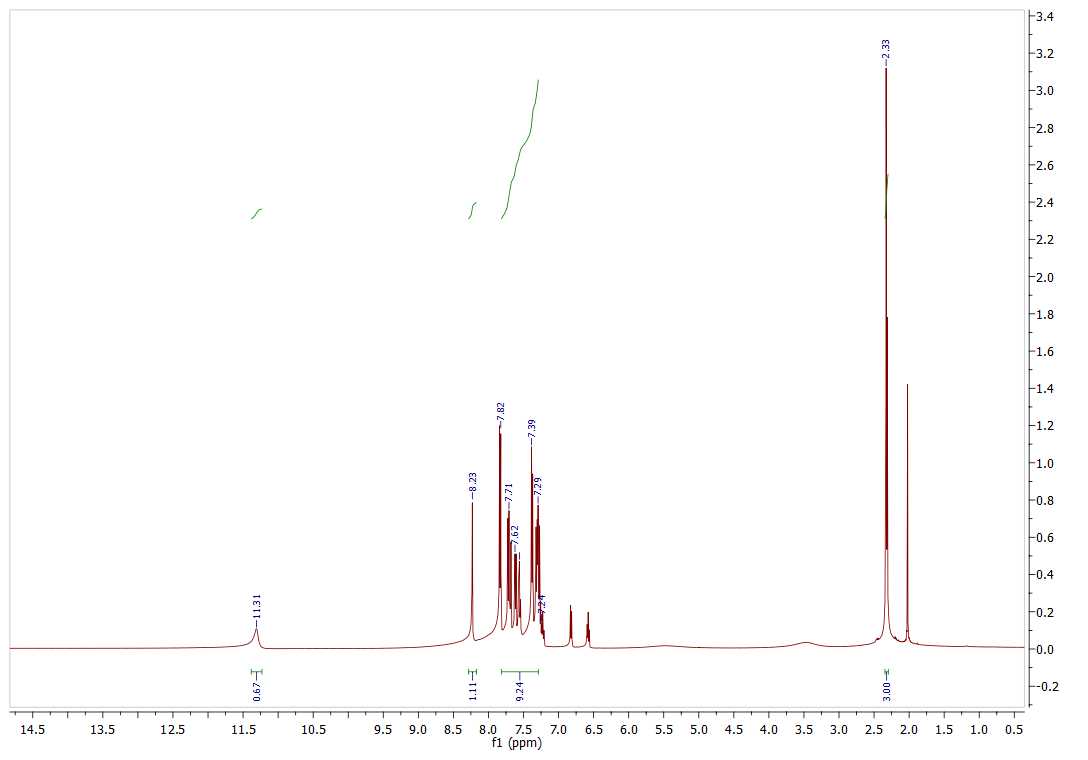
^

^1^H-NMR spectra of **11**

^
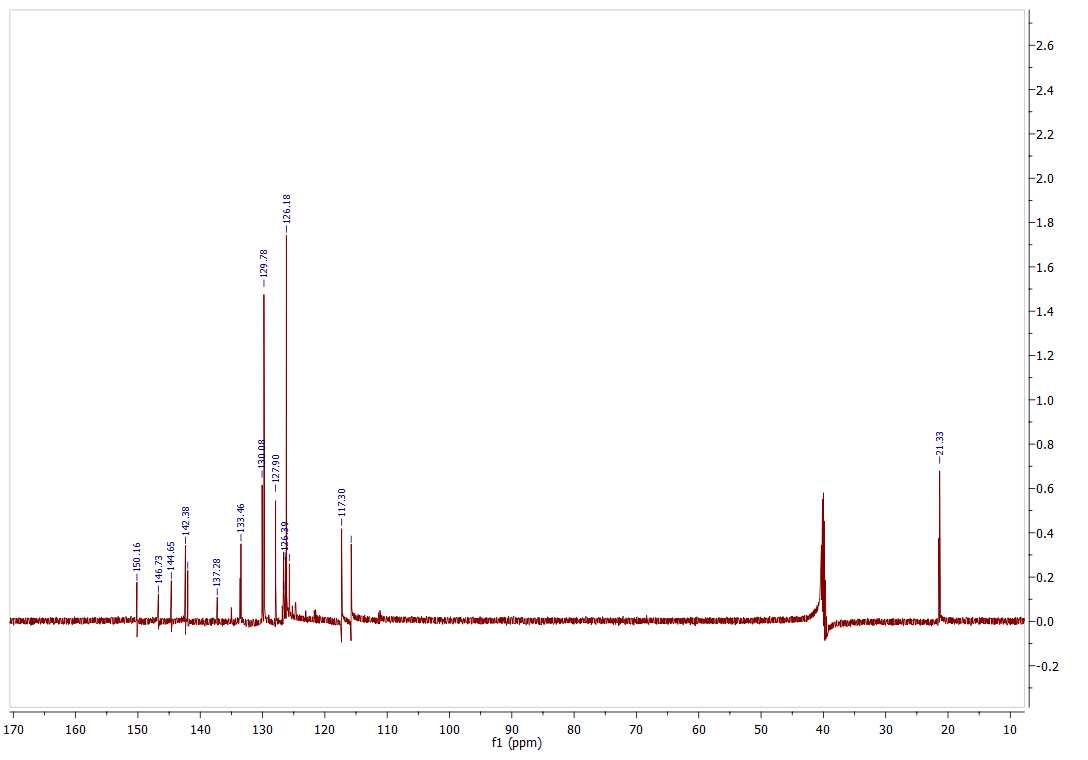
^

^13^C-NMR spectra of **11**


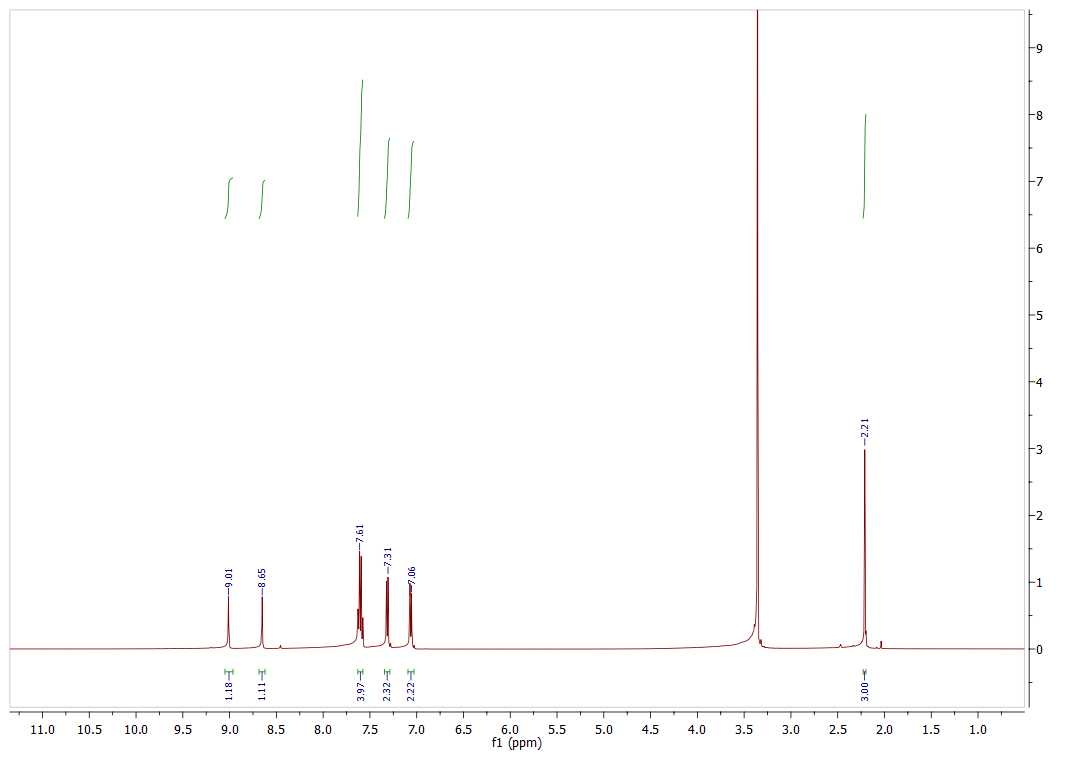


^1^H-NMR spectra of **13**


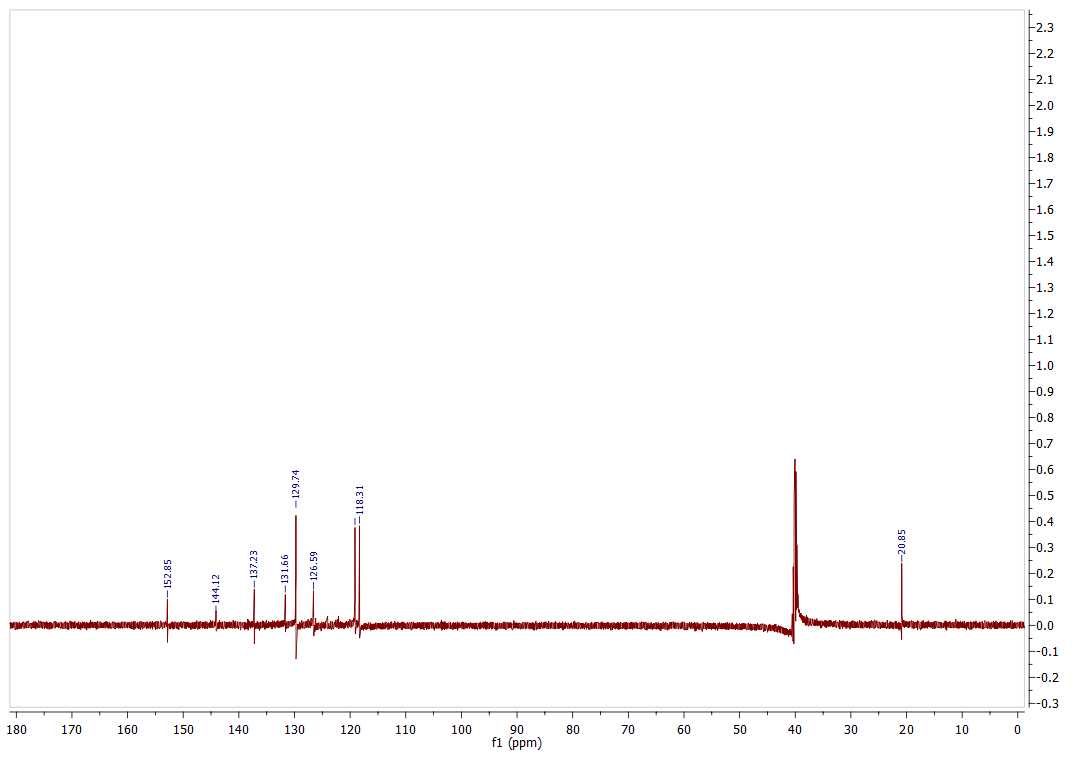


^13^C-NMR spectra of **13**


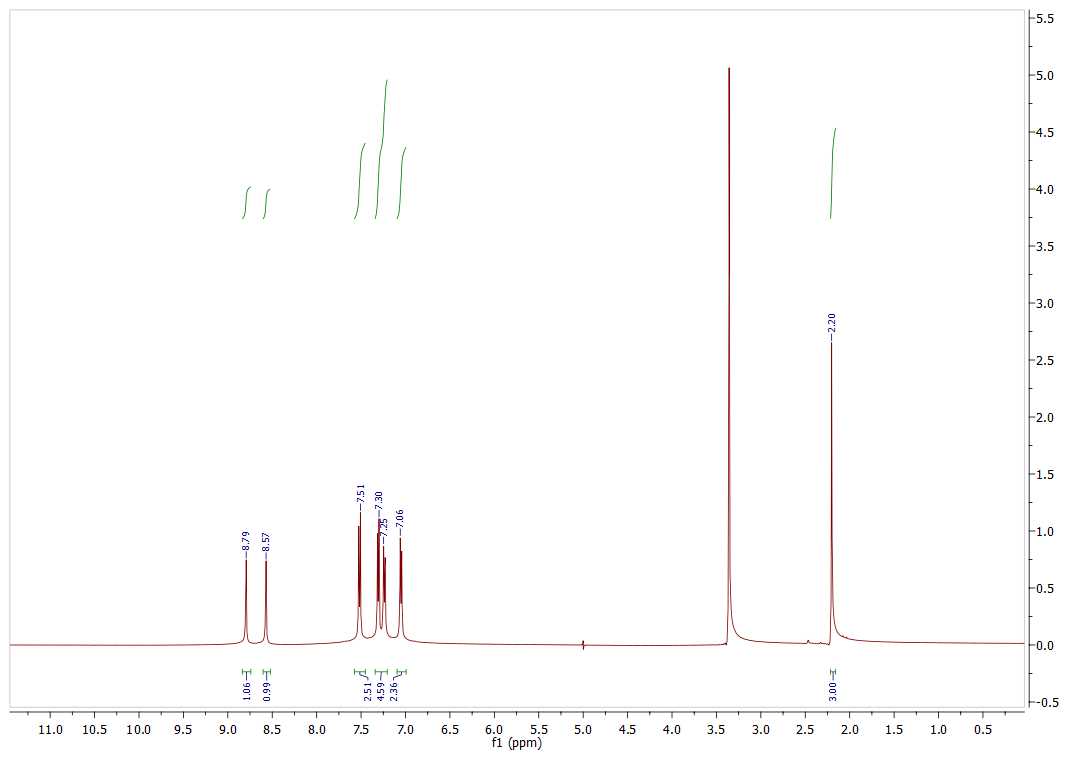


^1^H-NMR spectra of **14**


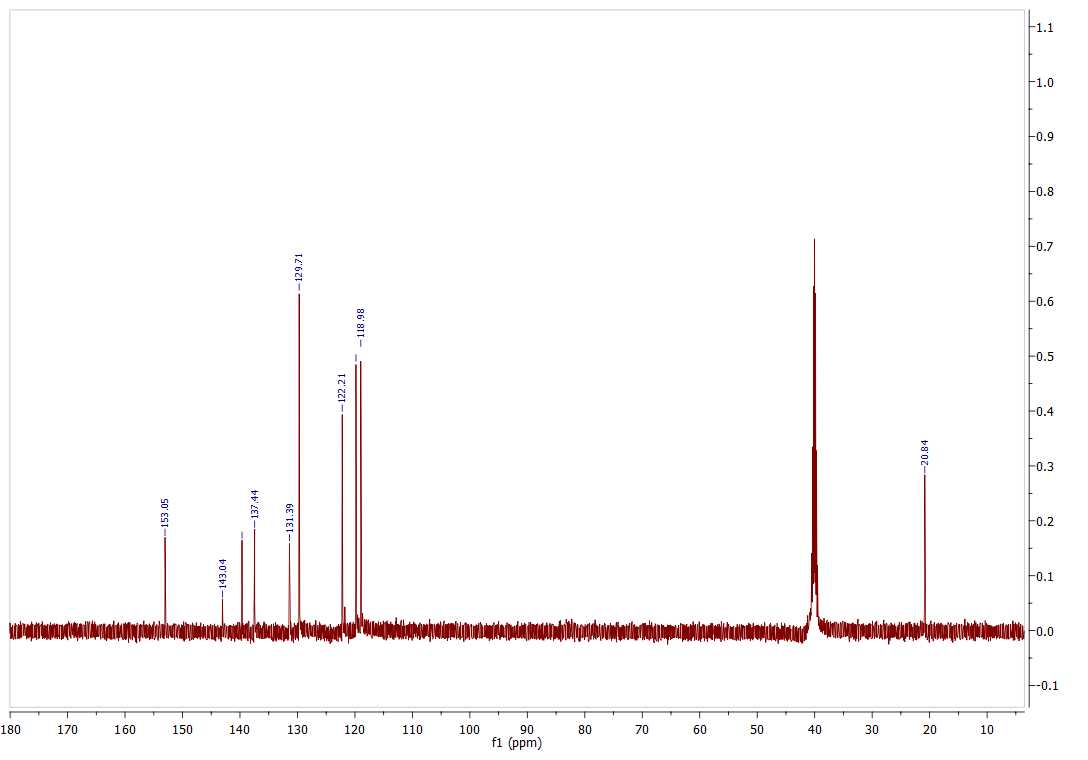


^13^C-NMR spectra of **14**

^
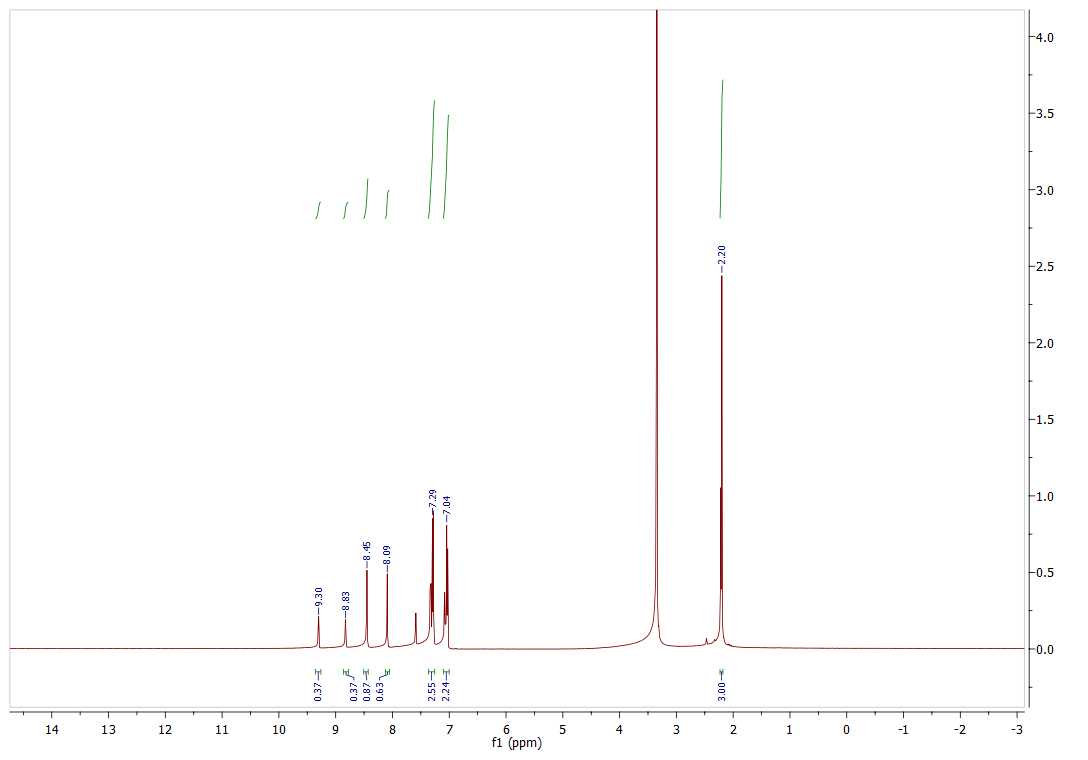
^

^1^H-NMR spectra of **15**


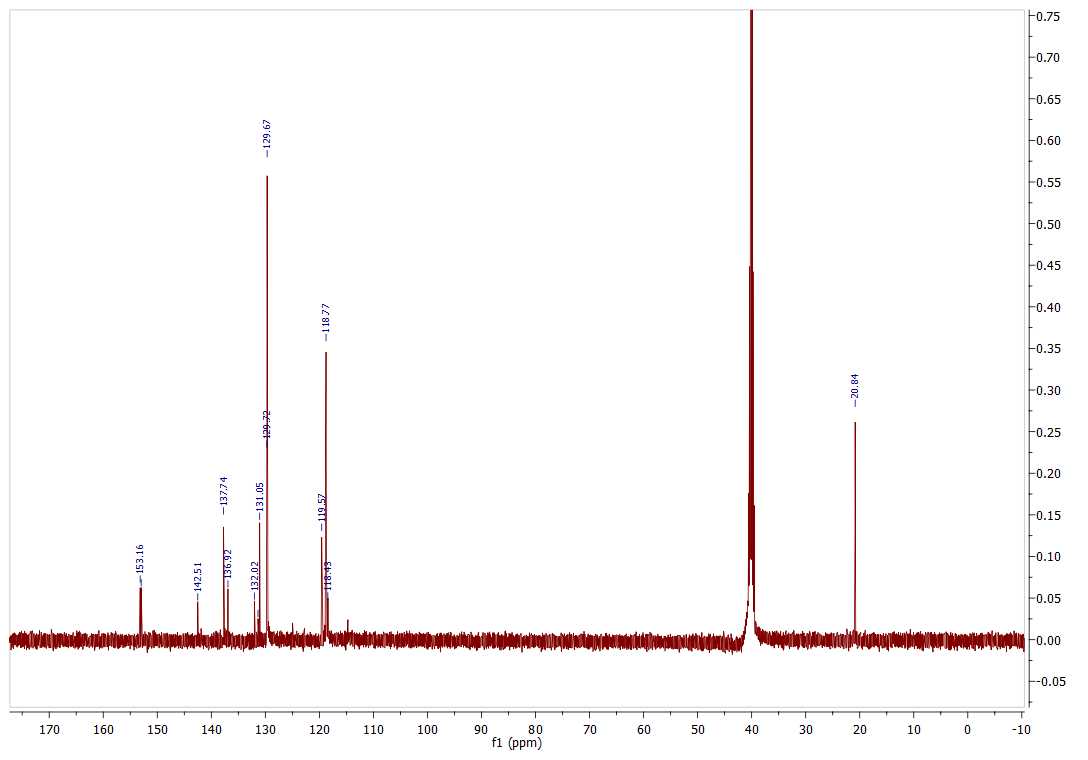


^13^C-NMR spectra of **15**


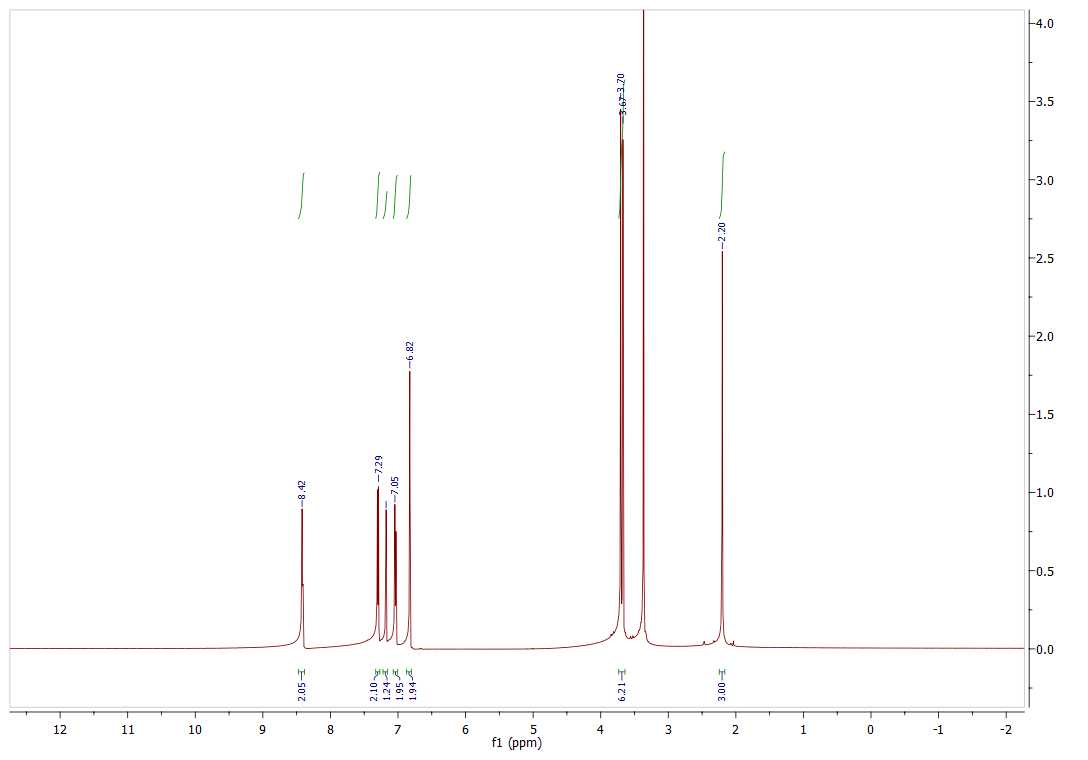


^1^H-NMR spectra of **16**


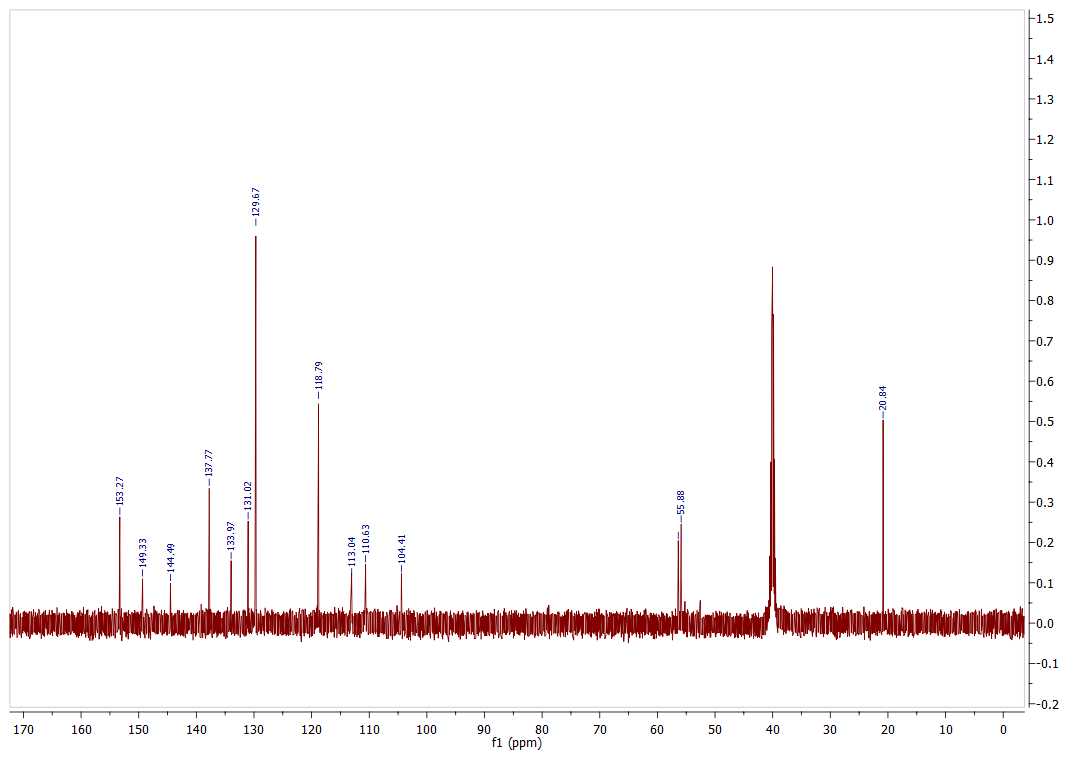


^13^C-NMR spectra of **16**


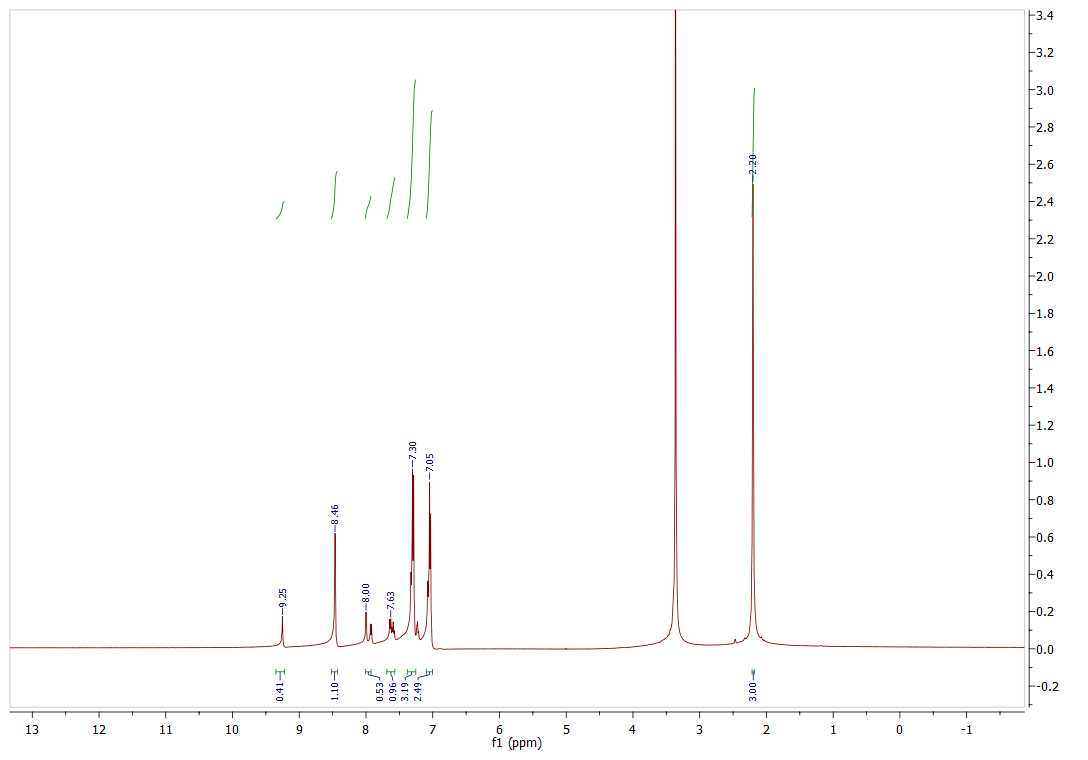


^1^H-NMR spectra of **17**


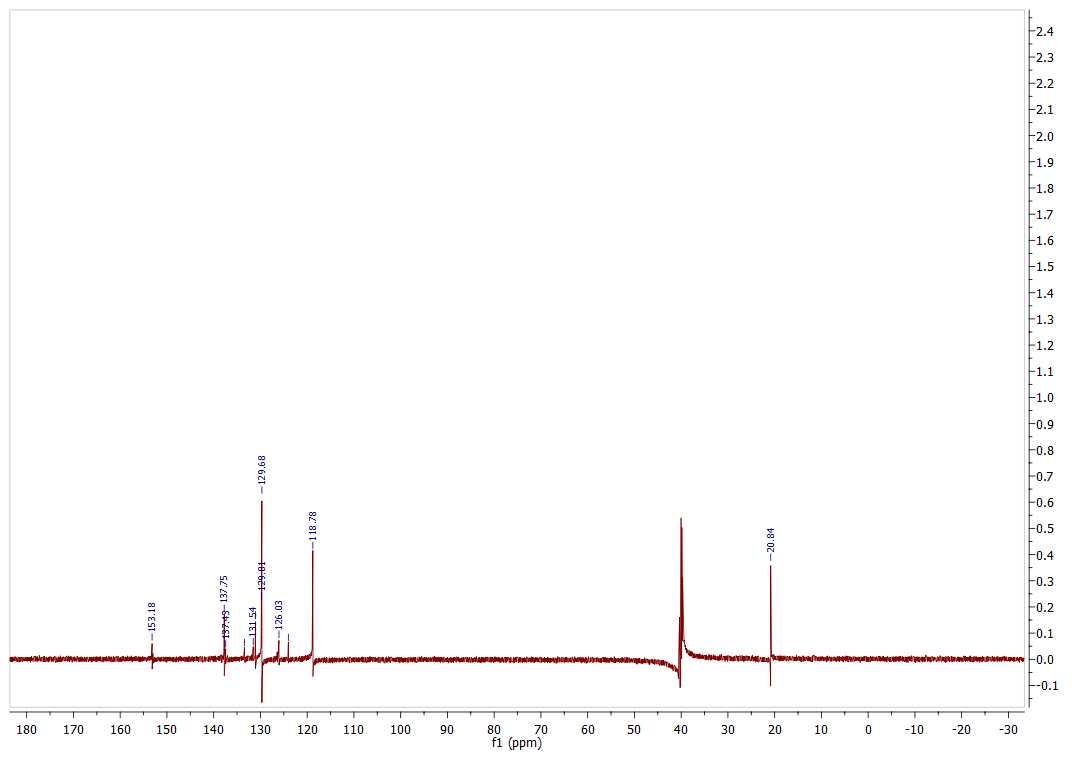


^13^C-NMR spectra of **17**

**Figure 4.** Alterations in the gene expression level of (a) *PALB2* gene in Paca2 cancer cell line treated with **7** and **15**. Data are presented as mean ± SEM. (a, b, c) Mean values within tissue with unlike superscript letters were significantly different (P<0.05).

**Figure 4.** Alterations in the gene expression level of (b) CDKN2A gene in Paca2 cancer cell line treated with **7** and **15**. Data are presented as mean ± SEM. (a, b, c) Mean values within tissue with unlike superscript letters were significantly different (P<0.05).

**Figure 4.** Alterations in the gene expression level of (c) *BRCA1* gene in PC3 cancer cell lines treated with **7**. Data are presented as mean ± SEM. (a, b, c) Mean values within tissue with unlike superscript letters were significantly different (P<0.05).

**Figure 4.** Alterations in the gene expression level of (d) *BRCA2* gene in PC3 cancer cell lines treated with **7**. Data are presented as mean ± SEM. (a, b, c) Mean values within tissue with unlike superscript letters were significantly different (P<0.05).

**Figure 5.** Alterations in the gene expression of (a) *EGFR* gene in A549 cancer cell line. Data are presented as mean ±SEM. (a, b, c) Mean values within tissue with unlike superscript letters were significantly different (P<0.05)

**Figure 5.** Alterations in the gene expression of (b) *KRAS* gene in A549 cancer cell line. Data are presented as mean ±SEM. (a, b, c) Mean values within tissue with unlike superscript letters were significantly different (P<0.05)

**Figure 5.** Alterations in the gene expression of (c) *TP53* gene in HCT116 cancer cells treated with **7** and **9**. Data are presented as mean ±SEM. (a, b, c) Mean values within tissue with unlike superscript letters were significantly different (P<0.05)

**Figure 5.** Alterations in the gene expression of (d) *FASN* gene in HCT116 cancer cells treated with **7** and **9**. Data are presented as mean ±SEM. (a, b, c) Mean values within tissue with unlike superscript letters were significantly different (P<0.05).

a)

M 1 2 3 4


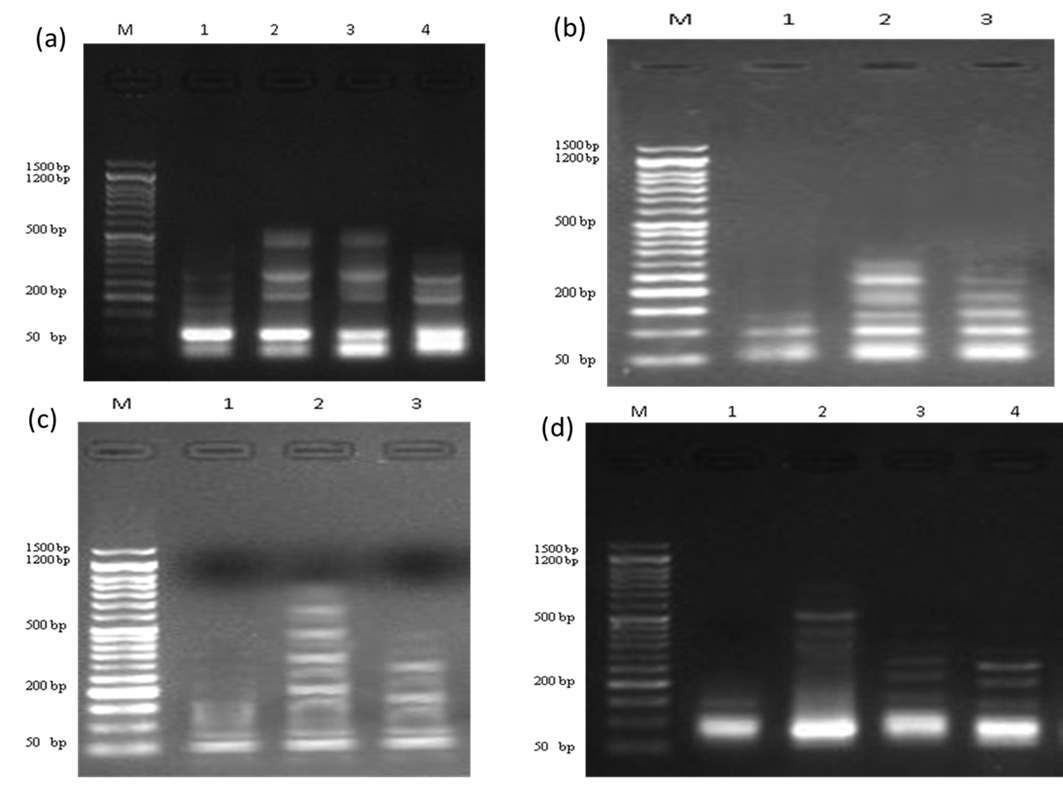


1500bp

1200bp

500bp

200bp

50bp

**Figure 7**. DNA fragmentation detected with Agarose gel in (a) PACA2 (lane 1: negative control, lane 2: **7**, lane3: **15**, lane 4: positive control); M: represent DNA marker.

b)

M 1 2 3


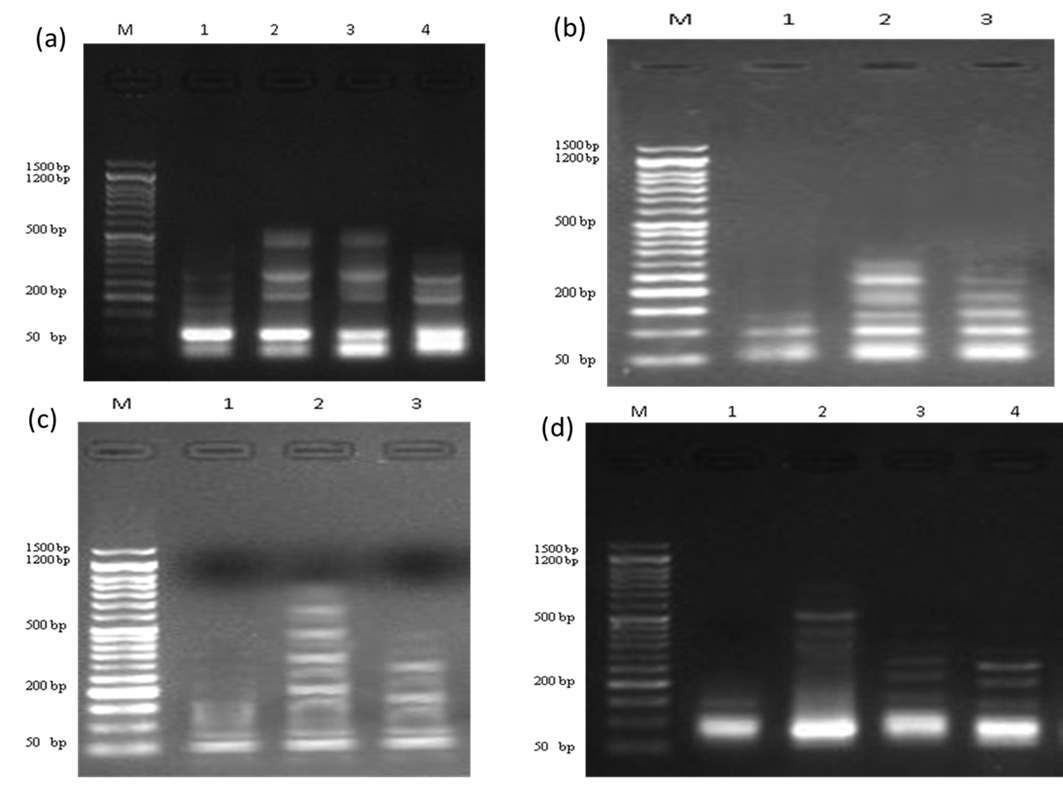
**
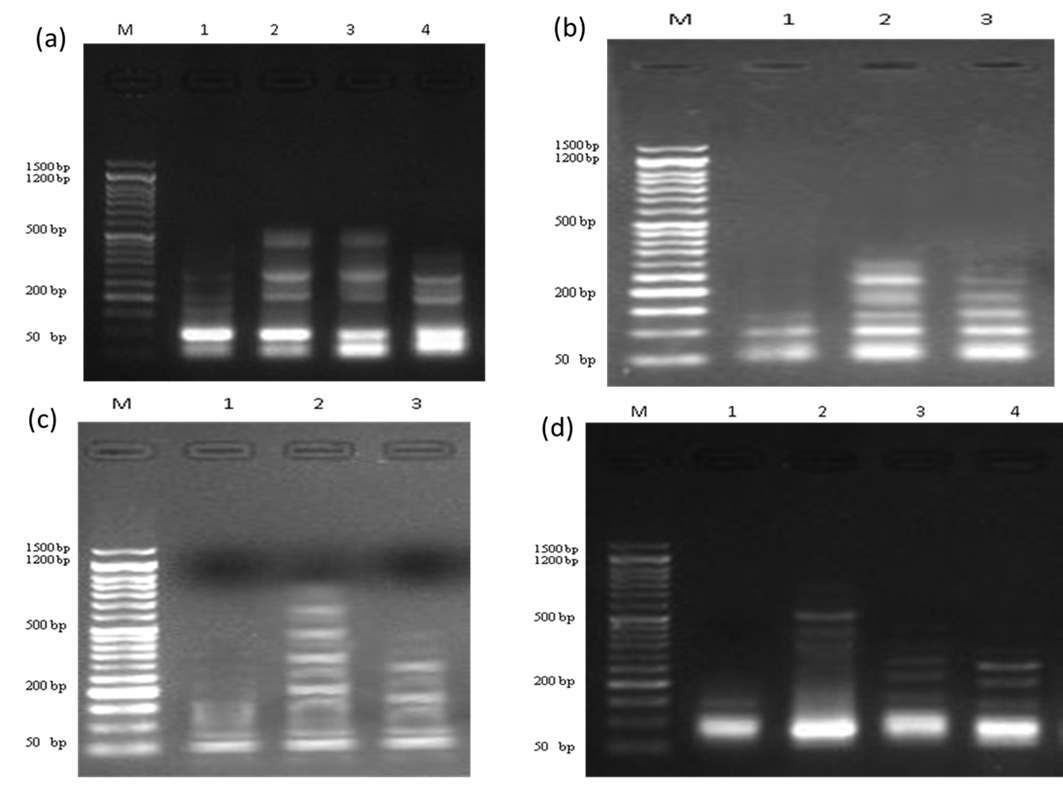
**

**Figure 7**. DNA fragmentation detected with Agarose gel in (b) PC3 (lane 1: negative control, lane 2: **7**, lane 3: positive control); M: represent DNA marker.

c)

**M 1 2 3**


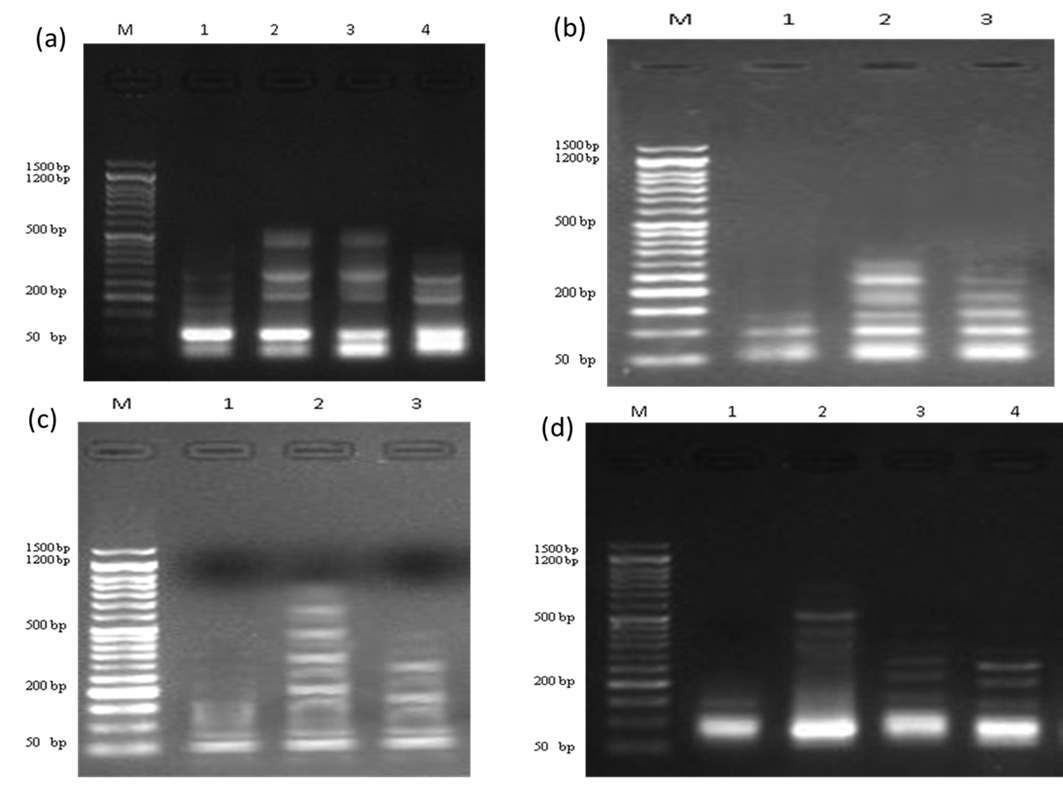

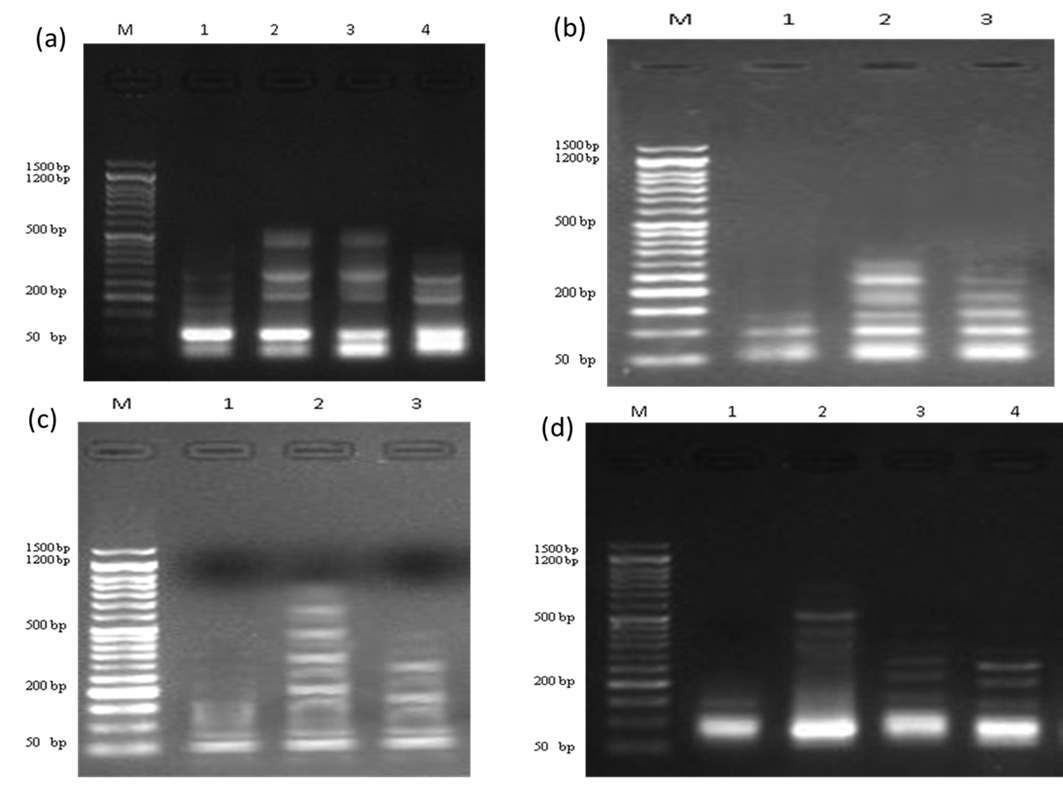


, **Figure 7**. DNA fragmentation detected with Agarose gel in (c) A549 (lane 1: negative control, lane 2 : **8**, lane 3: positive control); M: represent DNA marker.

d)

**M 1 2 3 4**


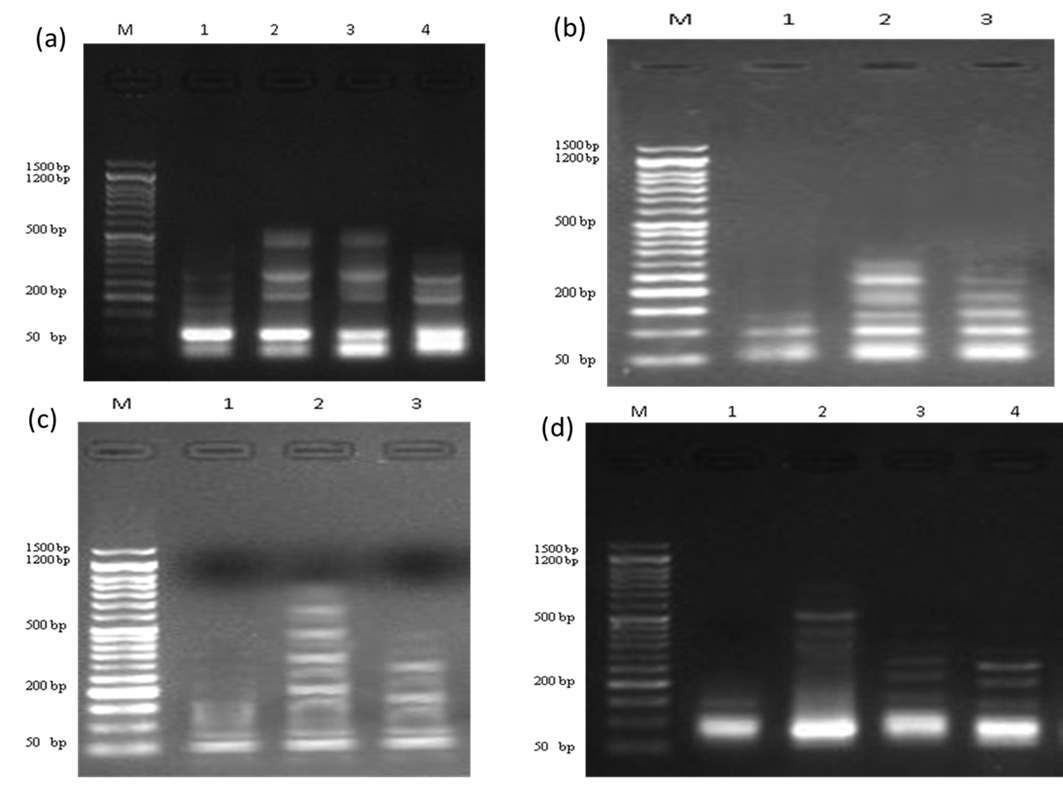

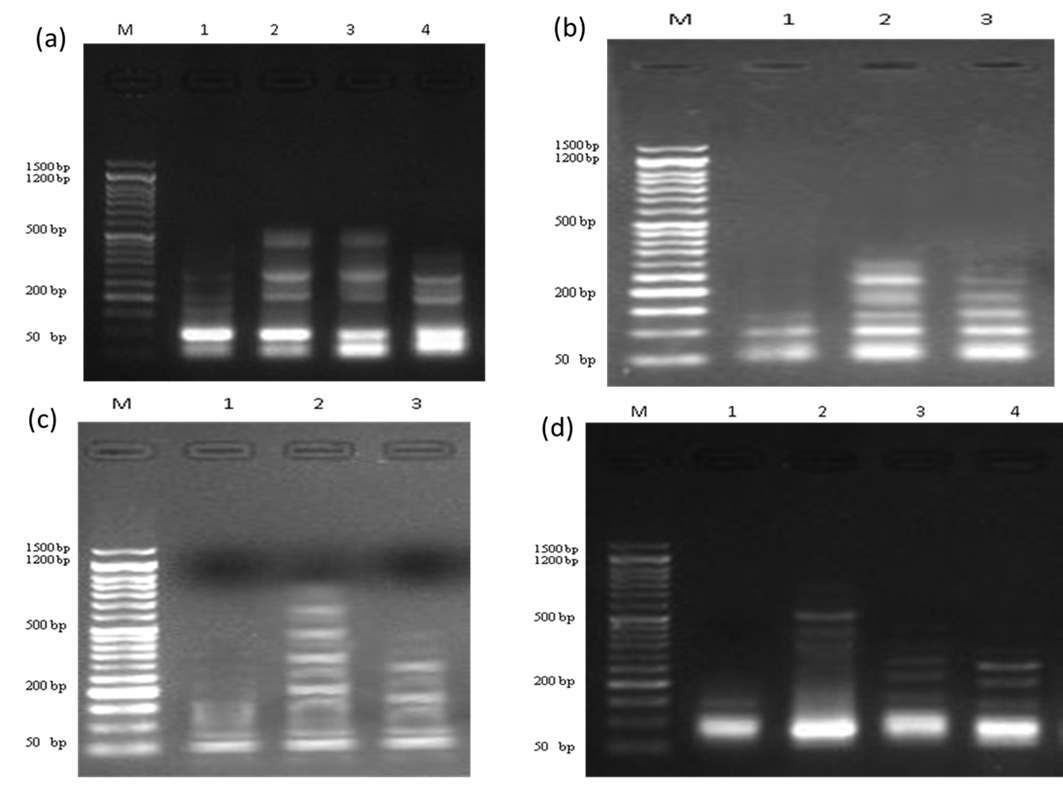


**Figure 7**. DNA fragmentation detected with Agarose gel in (d) HCT116 (lane 1: negative control, lane 2: **7**, lane 3: **9**, lane 4: positive control), M: represent DNA marker.


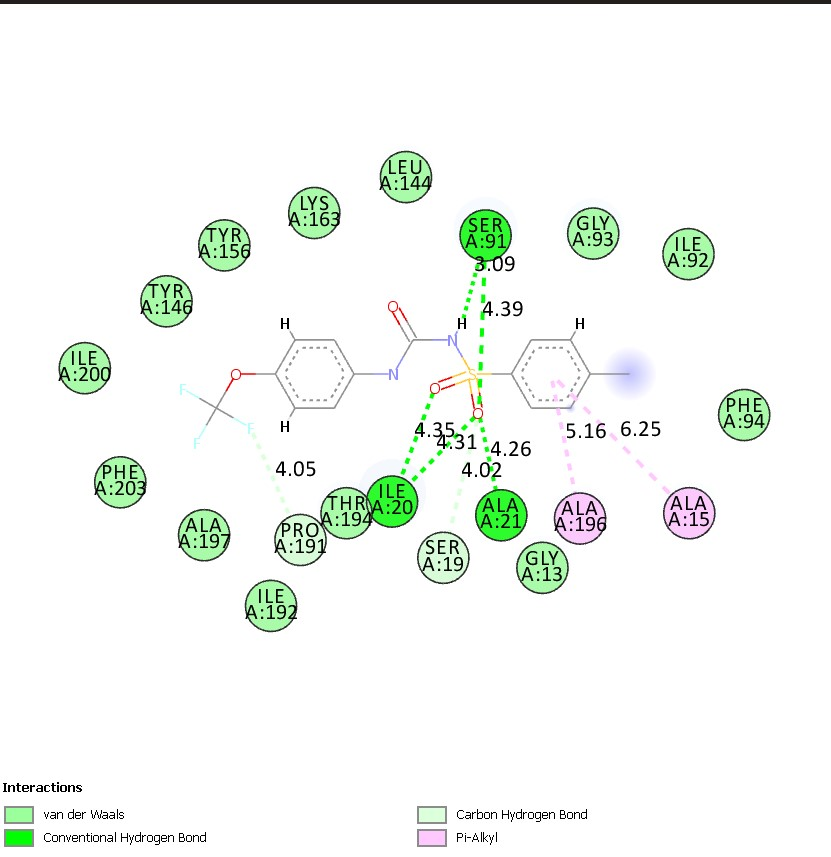

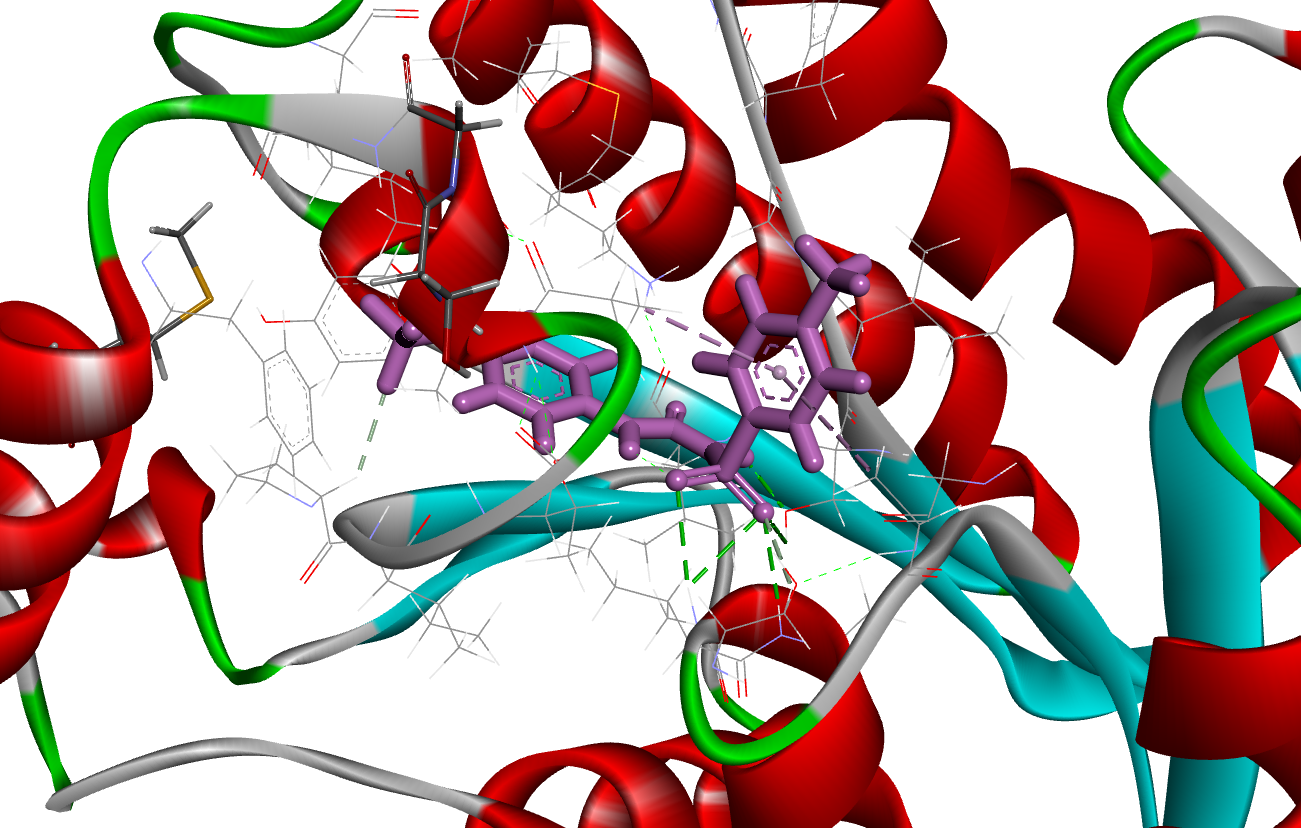


2D and 3D binding model between compound 8 and the active site of Escherichia coli enoyl reductase.


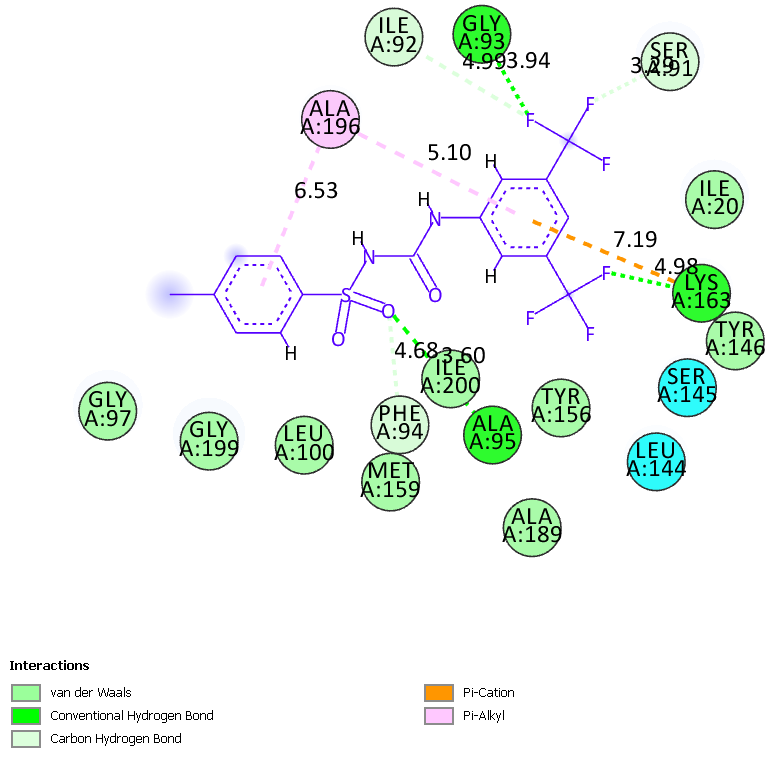

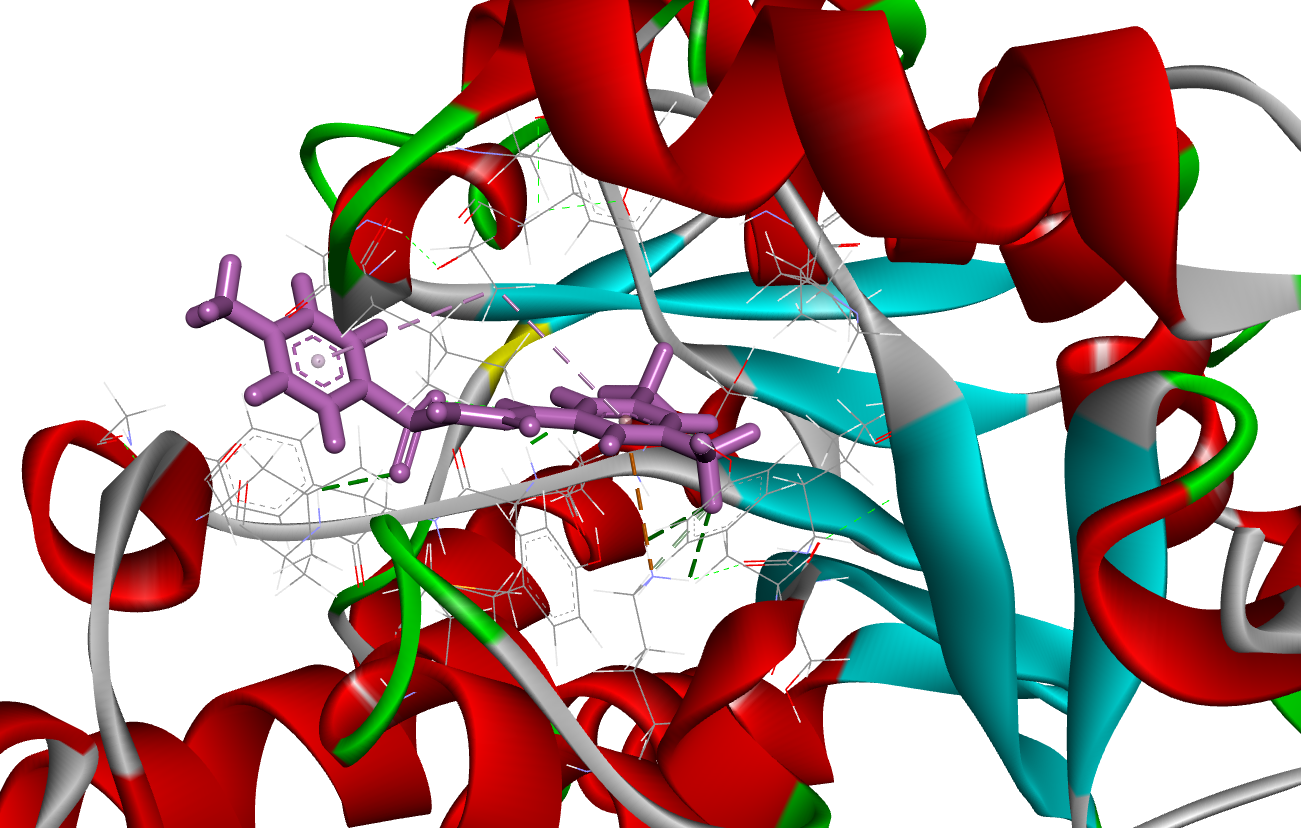


2D and 3D binding model between compound **9** and the active site of *Escherichia coli* enoyl reductase.


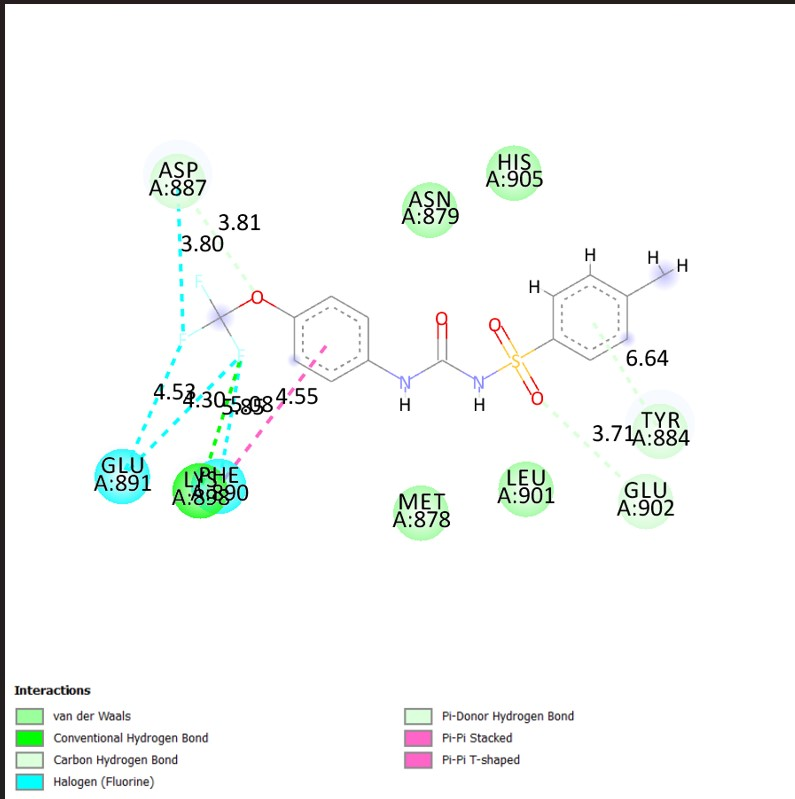

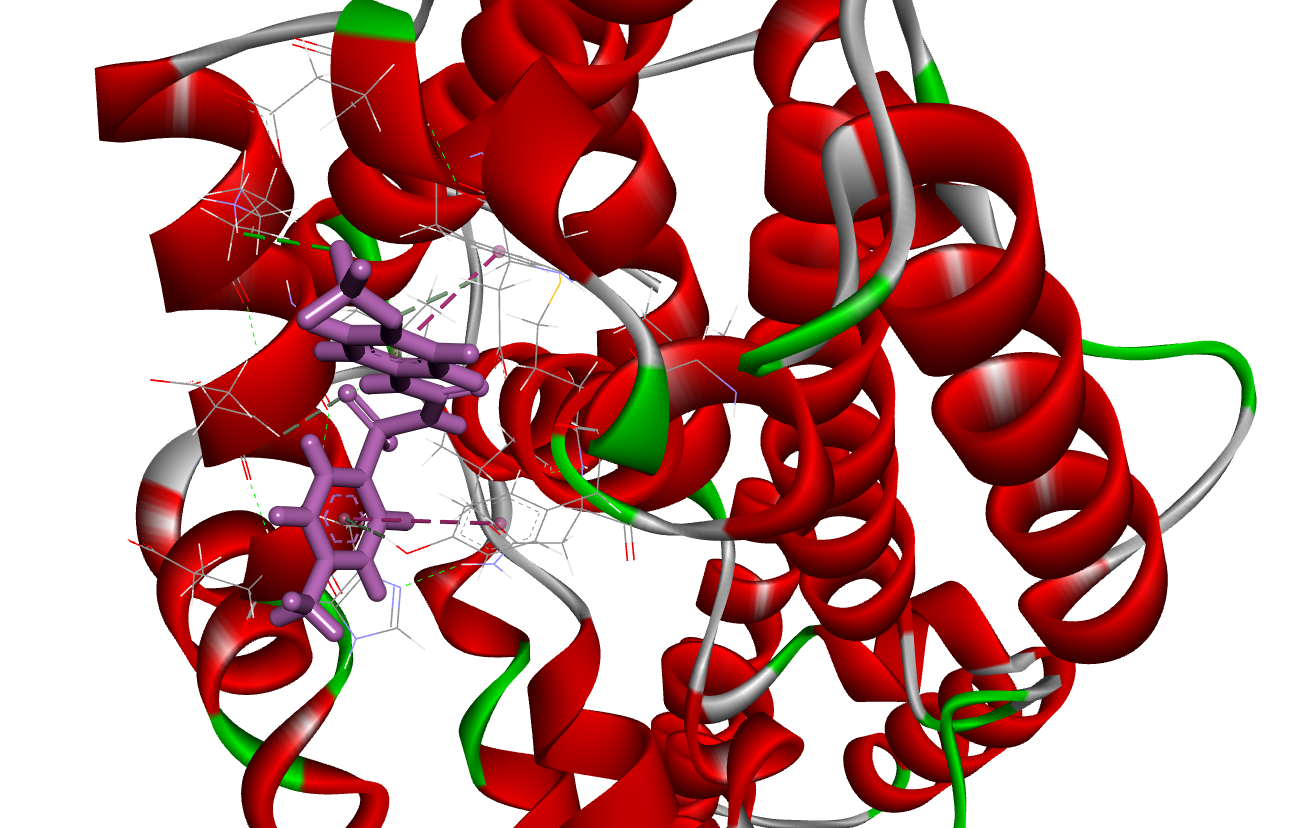


2D and 3D binding model between compound **8** and the active site of human SOS1.

**Figure 8.** The molecular interactions of compounds **8** and **9** with the active site of *E. coli* enoyl reductase and compound **8** with the active site of human SOS1
